# Design of an onboard computer for small experimental rockets with an integrated hardware-in-the-loop validation framework

**DOI:** 10.1016/j.ohx.2026.e00803

**Published:** 2026-06-16

**Authors:** Nathan Andreani Netzel, Leonardo Gabriel Rosa, Francisco Granziera, Marcelo Carvalho Tosin, Daniel Strufaldi Batista

**Affiliations:** State University of Londrina (UEL), Department of Electrical Engineering, Rod. Celso Garcia Cid– PR445, 86057-970, Londrina, PR, Brazil

**Keywords:** Onboard computer, Sounding rocket, MEMS sensors, Hardware-in-the-loop, Hardware validation

## Abstract

Small sounding rockets provide an accessible, cost-effective platform for education and experimental research, especially at universities. In such projects, the onboard computer (OBC) is essential to mission reliability. It is responsible for sensor data acquisition, real-time flight-state detection, data logging, and actuation during recovery events. This work presents the design and implementation of a dedicated OBC for small experimental rockets, together with systematic validation using an integrated hardware-in-the-loop (HIL) simulation framework. The proposed OBC integrates an ARM-based STM32F407 microcontroller, a multi-sensor measurement suite including inertial and barometric sensors, non-volatile data storage, dual pyrotechnic channels, a robust power-management subsystem, and a fully deterministic software architecture tailored for real-time flight-event detection. Complementing the flight hardware, the HIL environment reproduces the electrical, timing, and communication behavior of onboard sensors with high fidelity. Synthetic measurements derived from flight-dynamics simulations are injected through a dedicated interface, enabling end-to-end validation of data acquisition, state-transition logic, and onboard memory logging without requiring physical launches. Experimental HIL results demonstrate reliable detection of high-energy, low-energy, and apogee flight phases under realistic conditions, validating both the hardware design and algorithmic performance. The datasets and design files released with this work provide a reproducible foundation for educational and research activities in similar avionics projects.

## Graphical abstract

**Figure:**
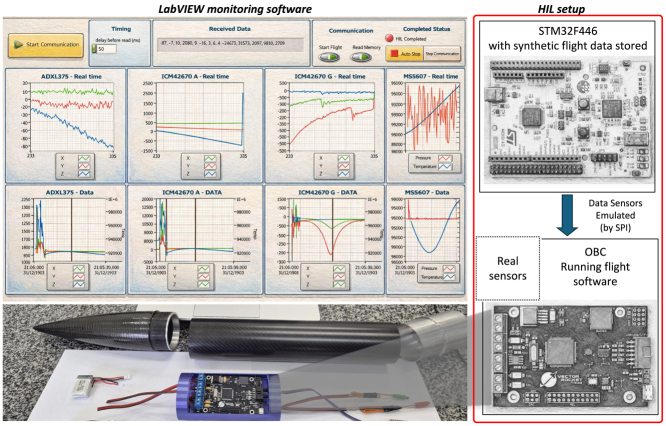

**Table:** 

Specifications table	

Hardware name	Onboard computer and a HIL validation framework for a small sounding rocket

Subject area	•*Engineering and material science*

Hardware type	• Aerospace engineering • Electrical engineering and computer science

Closest commercial analog	Commercial analogues of the onboard computer hardware (without any validation framework) includes the Raven4 altimeter from featherweight altimeters and the TeleMini from Altus Metrum.

Open source license	Creative Commons Attribution-NonCommercial 4.0 (CC BY-NC 4.0) license

Cost of hardware	$74.64 USD (Onboard Computer) and $15.88 USD (NUCLEO-H533RE)

Source file repository	https://doi.org/10.17632/dgf6yfv6p9.2

	

## 1 Hardware in context

Sounding rockets are important platforms for suborbital research, supporting a wide range of scientific and space environment investigations. Historically, they have also played a crucial role in national space programs, serving as technological precursors and capacity-building instruments for more complex missions [1].

Beyond their scientific relevance, sounding rockets have a significant academic impact, particularly within university-level aerospace engineering programs [2], [3], [4], [5], [6], [7], [8], [9], [10]. Several national space agencies have established structured educational programs to support student-led rocket projects [10], [11], [12], providing an affordable framework for developing technical infrastructure and training highly qualified personnel. In this setting, university-based sounding rocket initiatives offer a uniquely multidisciplinary environment [13], [14], [15], requiring the integration of aerodynamics, structures, propulsion, guidance, navigation, control, telemetry, and recovery systems, while simultaneously exposing students and researchers to the safety requirements and validation challenges inherent to aerospace development.

Within this multidisciplinary and challenging context, the onboard computer (OBC) is a central, safety-critical subsystem. It is responsible for sensor data acquisition, real-time flight-state detection, onboard data logging, and the actuation of irreversible events such as recovery system deployment. Failures in this subsystem directly compromise mission success and, more importantly, operational safety, making its correct operation a primary requirement for any experimental rocket mission.

Because of its importance, the challenges in designing OBCs for experimental rockets extend those encountered in conventional embedded systems. These platforms must operate deterministically under high acceleration while typically relying on low-cost MEMS sensors that are subject to noise, bias, and drift. Moreover, many critical flight events—such as motor ignition and parachute deployment—are non-reversible and cannot be safely exercised during real launches. Consequently, laboratory-level validation becomes a fundamental requirement, particularly in academic environments where repeated flight campaigns are costly, time-consuming, and logistically complex.

Historically, OBCs in amateur and experimental rocketry evolved from simple barometric altimeters to increasingly integrated avionics platforms. In the mid-1990s, Richard Osborne compiled a detailed comparison of the main flight computers available at the time [16]. Representative systems included the ALLTAC by Black Sky Research, the IAX-96 by Emmanuel Avionics, the FCB-M2 by Olsen, and the R-DAS by AED. These devices typically relied on analog barometric sensors, such as the MPX4100, for altitude estimation, and on axial accelerometers, such as the ADXL150, for longitudinal acceleration measurement. A feature considered advanced at that time was the ability to record flight data in non-volatile memory for post-flight analysis—now regarded as a basic capability, but then representing a significant technological milestone.

At present, commercially available OBCs reflect substantial advances in integration, sensing accuracy, processing capability, and telemetry. Widely used systems include the RRC3 by Missile Works [17], the StratoLoggerCF by PerfectFlite [18], the Raven4 by Featherweight Altimeters [19], and the TeleMini by AltusMetrum [20]. These platforms employ modern digital sensors, increased onboard storage capacity, configurable recovery logic, and, in some cases, real-time telemetry communication. Their reliability and maturity have made them standard tools in high-power and experimental rocketry.

Despite this technological evolution, the validation methodologies of such systems are predominantly based on practical flight campaigns. While flight testing is indispensable, it is inherently costly, operationally constrained, and limited in reproducibility during early development stages. In contrast to aerospace and automotive industries, where hardware-in-the-loop (HIL) simulation is widely adopted, the systematic application of HIL frameworks to the development and validation of onboard computers for small academic rockets remains sparsely documented in the open scientific literature.

HIL simulation is an effective validation methodology for the development of embedded systems for aerospace applications, such as experimental rockets, where extensive verification is required before real deployment. By integrating real hardware with simulated environments, HIL enables the complete sensing, processing, and decision-making chain to be exercised under realistic operating conditions before field implementation. This approach allows controlled stress testing of algorithms, repeatable evaluation of edge cases, and verification of deterministic behavior without exposing the vehicle to launch risks.

Therefore, this work addresses a gap between commercially flight computers and academically documented validation methodologies by presenting both the design of a dedicated onboard computer for small experimental rockets and a complete HIL-based validation framework conceived as an integral part of the hardware architecture. The proposed solution integrates custom avionics hardware, deterministic embedded software, and a sensor emulation environment capable of reproducing the electrical, timing, and communication behavior of real flight sensors. The embedded computer module is built around an STM32F407 microcontroller (ARM Cortex-M4 core). It supports multi-sensor acquisition (accelerometer, gyroscope, barometric pressure, and magnetometer), non-volatile flight-data logging, and dual pyrotechnic-channel control.

Unlike approaches that treat HIL as an auxiliary testing tool, the onboard computer presented here was designed from the outset to support end-to-end HIL validation via a dedicated external interface that exposes sensor communication buses and control lines. This design philosophy enables systematic laboratory-based verification before launch campaigns.

The system is demonstrated in the context of the Vector II project, a university-level initiative to establish experimental infrastructure for education and research in sounding rockets and aerospace systems. In this project, the primary mission is not a specific scientific payload, but rather the development and validation of onboard avionics and supporting test infrastructure that can be reused and adapted for future missions. The rocket platform thus serves as a representative application case for exercising the proposed OBC and HIL framework under realistic mission profiles.

By combining a dedicated avionics module with a reproducible HIL simulation environment, this work provides a practical and open framework for the development, testing, and validation of onboard computers in academic rocket programs. The approach enables systematic verification of hardware integrity, embedded software behavior, and mission logic without reliance on repeated flight tests, thereby reducing risk, cost, and development time while supporting education-oriented and research-driven aerospace initiatives.

## 2 Hardware description

Small experimental sounding rockets impose a distinct set of requirements on onboard electronic systems. Although mechanically simple when compared to orbital launch vehicles, these platforms operate under severe dynamic conditions, including high longitudinal acceleration, vibration, transient thermal loads, and limited opportunities for in-flight intervention. Consequently, the onboard computer has a central and safety-critical role, as it concentrates sensing, decision-making, data logging, and actuation of irreversible events such as recovery-system deployment.

From a hardware perspective, the role of the onboard computer is to interface reliably with inertial and environmental sensors, execute deterministic flight-state detection algorithms in real time, store mission data under constrained resources, and control pyrotechnic loads with high reliability. These functions must be performed using commercial of-the-shelf (COTS) components, typically MEMS-based sensors and microcontroller platforms, while maintaining predictable timing behavior and robustness against noise, disturbances, and component variability.

Importantly, the design of the hardware presented in this work was not conceived in isolation, but as the physical foundation required by a flight software of a small rocket. To detect all critical flight events, a minimal of longitudinal acceleration and barometric pressure measurements are required. Based on this principle, a deterministic algorithm, reported in previous works [21] was implemented as part of the OBC. The algorithm must identify three key flight phases:

• **High-energy stage:** occurs immediately after engine ignition and is characterized by a sharp increase in acceleration as the engine provides thrust, rapidly increasing the vehicle’s velocity.
• **Low-energy stage:** occurs shortly after engine shutdown and is marked by a slight deceleration due to aerodynamic drag as the vehicle loses most of its kinetic energy.
• **Apogee detection:** determined when consecutive increases in barometric pressure are observed, indicating a decrease in altitude. This event triggers deployment of the parachute system. For high-performance configurations, the hardware supports dual-stage recovery, firing drogue and main parachutes at predefined altitudes.

The selection of sensors, communication protocols, and acquisition rates was driven by the need to execute filtering and flight-state detection algorithms deterministically in real time. At the architectural level, the hardware was conceived to support hardware-in-the-loop validation natively, ensuring that sensor interfaces, signal accessibility, and communication paths allow realistic stimulation and observation of the onboard system. This architectural support enables laboratory-level testing of both the embedded software and the developed hardware under representative flight conditions. As a result, the complete acquisition, processing, and decision chain can be validated prior to flight, increasing robustness against noise, timing uncertainties, and implementation-level faults.

To provide contextual grounding for the proposed avionics system, the following subsection briefly describes a representative small sounding rocket structure and its minimal subsystems. This description is intended solely to frame the operational environment and interface requirements of the onboard computer; the present work does not aim to document or validate the mechanical or propulsion design of the rocket itself. Subsequent subsections then focus exclusively on the onboard computer hardware, the embedded software architecture, and the HIL simulation framework.

### 2.1 A typical small rocket structure and subsystems description

**Fig. 1:**
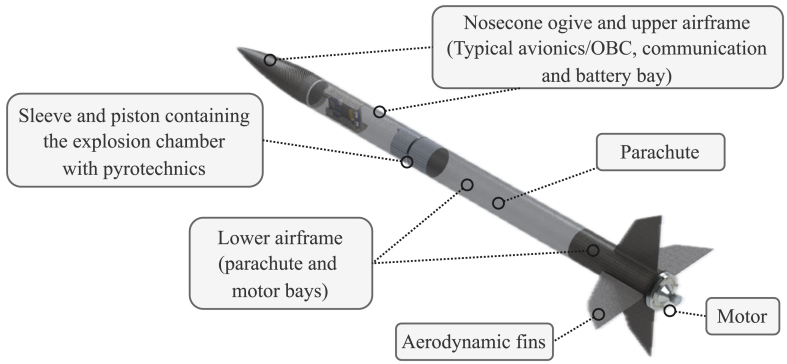
Representative image of a typical rocket structure and its subsystems.

Fig. 1 illustrates a representative small, university-scale sounding rocket, used here as a reference platform to contextualize the operating environment of the onboard computer. Such vehicles are typically characterized by compact dimensions, low launch mass, and modular construction, enabling repeated assembly, testing, and recovery within academic programs.

From a system perspective, a small experimental rocket can be decomposed into a limited set of functional subsystems that interact directly or indirectly with the onboard electronics:

• **Structural airframe** — provides the mechanical backbone of the vehicle and defines the available volume for avionics, recovery systems, and propulsion components;
• **Propulsion subsystem** — responsible for generating thrust during a short burn interval, imposing high acceleration and vibration loads on the onboard electronics;
• **Recovery subsystem** — typically based on parachutes or similar devices, whose deployment is commanded by the onboard computer through pyrotechnic or electromechanical actuators;
• **Power subsystem** — supplies energy to avionics and actuation circuits, often through independent domains for logic and pyrotechnic loads;
• **Communication subsystem** - enables umbilical and telemetry and telecommand communications.
• **Onboard computer (avionics)** — interfaces with sensors, executes real-time flight-state detection algorithms, logs mission data, controls recovery events, and manages communication.

Although the detailed mechanical design of these subsystems varies across projects, the functional interfaces between them remain largely consistent in small academic rockets. In particular, the onboard computer must operate reliably while subjected to the mechanical disturbances generated by the propulsion phase and must remain electrically and logically stable during recovery deployment and descent.

In the context of the present work, the rocket platform serves as an application example that defines the environmental and interface constraints of the avionics system. The mechanical structure, propulsion design, and recovery hardware are therefore described only at a conceptual level, sufficient to motivate the requirements imposed on the onboard computer and its validation framework. Detailed analysis and validation of the rocket structure itself are outside the scope of this article.

The following subsections focus exclusively on the onboard computer developed to operate within this environment, detailing its hardware architecture, embedded software organization, and the hardware-in-the-loop simulation framework used for systematic validation.

### 2.2 Onboard computer hardware description

**Fig. 2:**
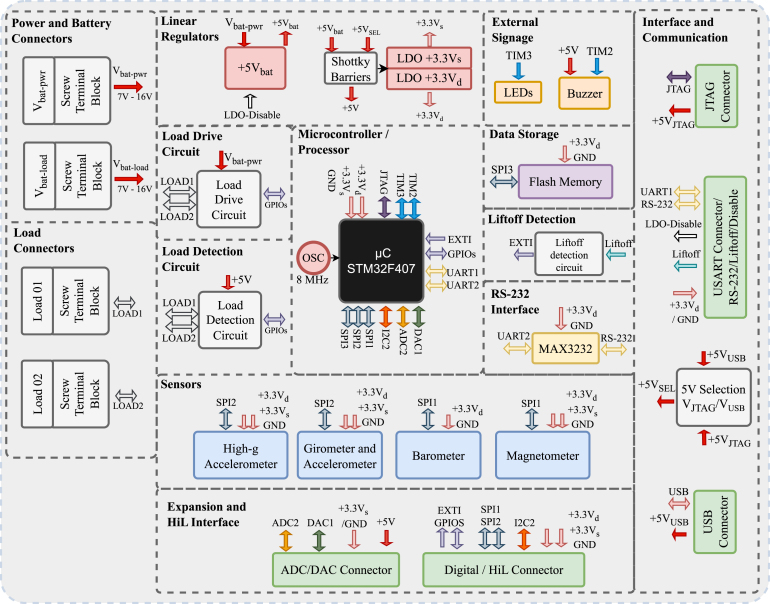
General diagram of the rocket’s hardware.

The onboard computer presented in this work is a custom system designed for small-scale experimental sounding rockets and demonstrated within the Vector II project as a representative application case. Unlike development-board-based solutions commonly adopted in academic rockets, which often rely on multiple peripheral modules and extensive wiring, the proposed design integrates sensing, processing, data storage, power distribution, and actuation functions into a single, compact, mission-oriented printed circuit board (PCB). This high level of integration increases reliability, reduces interconnection failures, and establishes a deterministic embedded platform suitable for real-time flight operations. A system-level overview of the hardware is shown in Fig. 2, which illustrates the major functional blocks and their interfaces.

At a high level, the onboard computer is composed of five main subsystems: (i) a processing unit based on an STM32 microcontroller, (ii) a suite of digital sensors for acceleration, angular rate, pressure, and magnetic-field measurements, (iii) non-volatile data storage for flight logging, (iv) power regulation and pyrotechnic actuation circuitry, and (v) external interfaces for communication and system integration. These subsystems were co-designed to meet the deterministic timing requirements imposed by the flight-phase detection algorithm discussed in Section 2. Although validated within the Vector II platform, the hardware architecture was conceived to be reusable across different small experimental rocket configurations with minimal adaptation.

At the core of the system is the **STM32F407VGT6** microcontroller from STMicroelectronics [22], featuring an ARM 32-bit Cortex-M4 CPU with floating-point unit (FPU) running at up to 168 MHz. Its 1 MB of Flash memory and 192 kB of SRAM provide sufficient resources for deterministic execution of filtering routines, event-detection logic, and data logging. The microcontroller also offers multiple SPI, I^2^C, and USART/UART interfaces, enabling seamless integration of high-speed digital sensors and peripherals. Its computational performance, mature toolchain support, open-source development ecosystem, and the research group’s prior experience with STM32 platforms motivated its selection.

Sensor acquisition is organized around four digital devices connected through dedicated SPI buses to guarantee low-latency and synchronized sampling, as highlighted in blue in Fig. 2. Although only longitudinal acceleration and barometric pressure are strictly required for deterministic flight-phase detection [21], additional sensors were included to provide redundancy and support future attitude-estimation developments. The **ADXL375** accelerometer [23], interfaced via SPI2, provides high-range acceleration measurements up to ±200g, enabling reliable detection of extreme dynamic events such as motor ignition and burnout. This wide measurement range is critical, as propellant-characterization experiments indicated that acceleration levels during the high-energy stage may exceed the ±16g limit of the ICM-42670-P inertial measurement unit (IMU) [24]. Sharing the same SPI2 bus, the **ICM-42670-P** IMU [25] supplies three-axis gyroscope and accelerometer data with higher resolution, complementing the ADXL375 for redundancy and potential attitude estimation. Altitude information is obtained from the **MS5607** barometric pressure sensor [26] on SPI3, which provides compensated pressure readings and temperature data for calibration and post-flight analysis. Finally, the **MMC5983MA** magnetometer [27], connected via SPI1, measures the local magnetic field with an ±8G full-scale range and 18-bit resolution, supporting future studies on magnetic-based attitude estimation. Together, these sensors form a comprehensive and extensible measurement suite that exceeds the minimal requirements for event detection while remaining aligned with mission scalability.

To ensure precise timing behavior, each sensor is assigned an independent chip-select line and a dedicated interrupt pin routed directly to the microcontroller. This configuration enables deterministic sampling and precise synchronization across all data channels, ensuring that acceleration and pressure data are processed reliably and with minimal temporal uncertainty.

Non-volatile flight data storage is provided by an **AT45DB641E** serial flash memory [28], represented in purple in Fig. 2. Connected through a dedicated SPI4 bus, the 64 Mbit device supports high-speed logging of raw sensor data and algorithm outputs. The use of an independent SPI channel ensures that logging operations do not interfere with sensor acquisition or other timing-critical tasks, preserving deterministic behavior even during high-frequency sampling.

The board also integrates dedicated circuitry for power distribution, signal routing, and pyrotechnic actuation. Two independent pyrotechnic channels support drogue and main parachute deployment, enabling dual-stage recovery configurations. Each channel includes activation and continuity-monitoring circuits that allow verification of squib integrity prior to firing. Load activation is performed using a solid-state MOSFET stage based on the **IRF7103PBF**, selected over mechanical relays to avoid failures associated with vibration and high inertial loads during launch. Transient-protection components and isolation resistors further protect sensitive electronics from current spikes and electromagnetic interference. Additional circuit-level details are provided in Section 5.

The power-supply architecture was designed to ensure robust and stable operation throughout all mission phases. Power is distributed across two independent supply domains, each with its own connector and battery: one dedicated to the avionics electronics and another to the pyrotechnic loads. Although powered by separate two-cell (2s) Li-ion batteries (7.4V), both domains share a common ground reference to ensure consistent signal levels across the system. The batteries operate over a 7–16V discharge range. In the electronics domain, a high-current **MIC29302WU** low-dropout regulator (LDO) [29] generates a stable +5V rail. From this rail, two low-noise **LP5907** regulators [30] generate independent +3.3V supplies: one dedicated to the digital circuitry, including sensor digital interfaces, and another exclusively powering the analog supply rails of the sensors. This separation minimizes coupling of digital switching noise into the analog measurement paths, improving sensor precision. The pyrotechnic domain is powered directly from its dedicated battery to allow high-current delivery without inducing voltage drops in the avionics supply rails. Terminal block connectors are used for power and pyrotechnic connections to ensure robust mechanical fixation under vibration and acceleration loads.

During ground tests, the board may alternatively be powered through the Micro-USB or DEBUG connectors via a Schottky-diode source-selection circuit, facilitating integration with Electrical Ground Support Equipment and the hardware-in-the-loop setup. Visual indicators in the form of LEDs provide feedback for each voltage rail, and a resistive divider connected to one of the microcontroller’s ADC channels enables real-time monitoring of the battery voltage. Further details regarding regulator selection, component sizing, and voltage-monitoring circuitry are provided in Section 5.

A set of well-defined external interfaces, highlighted in green and white in Fig. 2, enables communication, programming, and integration with ground-support equipment. The Micro-USB connector supports power delivery, serial communication, and firmware updates, protected by a TVS diode and LC filter. The 10-pin ST-LINK/V2-Mini-compatible header provides Serial Wire Debug (SWD) access to the microcontroller.

The 12-pin connector exposes the RS-232 interface derived from USART2, the USART1 TTL RX and TX pins, and two auxiliary control lines: the LIFTOFF_READ signal, used to detect rail separation, and the DISABLE line, which remotely deactivates the +5V regulator during pre-launch operations. These are intended to interface with an external Electrical Ground Support System (EGSS) during mission preparation and launch operations. Through this interface, the onboard computer can exchange telemetry, receive arming or inhibit commands, provide firing-ready status signals, and allow remote health monitoring prior to liftoff. Such ground-support architectures are commonly employed in sounding-rocket and launch-vehicle operations to ensure safe integration and controlled activation of flight-critical subsystems.

Although the complete implementation of an EGSS is outside the scope of the present work, the hardware was designed to natively support this integration. During HIL operation, these connectors may optionally be used for additional monitoring or command injection; however, their primary role is to enable safe and structured interaction between the onboard computer and ground systems during real mission scenarios. In addition, the availability of both RS-232 and UART TTL interfaces allows direct integration of wireless communication modules (e.g., RF or optical links) without modifications to the onboard hardware, enabling real-time telemetry and remote monitoring through a simple external interface.

To support ground testing and in-flight status monitoring, the onboard computer includes four status LEDs and a buzzer, highlighted in orange in Fig. 2. Driven directly by STM32 timer outputs, they provide precisely timed visual and acoustic signals conveying system states such as power availability, firmware activity, communication status, and fault detection.

The 20-pin *HIL support* connector provides direct access to all sensor communication interfaces and control signals, including SPI1–SPI4, I^2^C2, and additional GPIOs configured as chip-select and interrupt lines. This interface allows physical sensors to be replaced by simulated signals from Electrical Ground Support Equipment during hardware-in-the-loop testing. Through this connector, the onboard computer can operate under realistic mission conditions without requiring the complete flight hardware assembly, enabling deterministic verification of data-acquisition timing, filtering routines, and event-detection logic. The *HIL support* connector therefore plays a central role in the validation workflow described in Section 2.4, bridging the avionics hardware and the simulation environment. Lastly, the 6-pin ADC/DAC connector enables analog measurements and outputs for calibration, testing, and future expansion.

Overall, the proposed onboard computer integrates aerospace-grade sensors, a high-performance microcontroller platform, and an optimized PCB layout to ensure deterministic operation, electrical reliability, and high data integrity. By consolidating sensing, processing, data logging, and pyrotechnic control into a single mission-oriented PCB, the design delivers a robust and flight-ready avionics solution. The native support for hardware-in-the-loop validation further distinguishes the architecture, enabling end-to-end testing with Electrical Ground Support Equipment in a manner that would be significantly more complex when using general-purpose development boards. This integrated approach establishes a solid foundation for real-time telemetry, advanced state estimation, and repeatable laboratory validation in future academic rocket missions.

### 2.3 Overview of the OBC embedded software

The embedded software developed for the onboard computer follows a deterministic and modular architecture co-designed with the underlying hardware to ensure predictable timing across sensor acquisition, flight-state detection, data logging, and actuation. The implementation builds upon previous developments of the Vector Project related to deterministic flight-event detection algorithms [21], while extending the software architecture to support a broader sensor suite, onboard non-volatile data storage, and native HIL simulation capability.

The software is implemented in C using STMicroelectronics’ Hardware Abstraction Library (HAL) version 1.4.0. To maximize maintainability and reproducibility, each hardware component is abstracted through an independent driver implemented as paired .c and .h files and documented using the Doxygen standard. These drivers encapsulate sensor-specific details such as register maps, data formats, timing constraints, and conversion formulas, exposing a unified interface to the main application. This modular approach allows new sensors or hardware revisions to be incorporated with minimal changes to the core codebase.

Supporting these drivers, a set of callback routines handles UART reception, sensor *data-ready* interrupts, and flash-memory programming events. All time-critical operations are implemented in a non-blocking manner and rely on interrupt-driven or DMA-based transfers, ensuring predictable response times even during high-throughput acquisition phases.

**Fig. 3:**
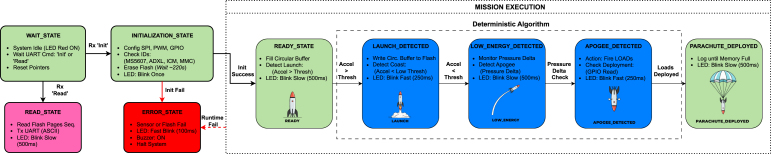
Overview of the finite state machine implemented in the onboard software. Mission states (green), event detection algorithm (blue), and postflight data readout (pink).

The core application logic is organized as a finite state machine implemented in main.c. This design provides a transparent and traceable execution flow, simplifying debugging, validation, and adaptation to future mission profiles. The state machine governs system initialization, nominal flight execution, data logging, fault handling, and post-flight data retrieval. Fig. 3 illustrates the main software states, highlighting mission states (green), the deterministic event-detection algorithm (blue), and the post-flight data-readout sequence (pink).

Upon system reset, the onboard computer enters the WAIT_STATE, where it remains idle until a serial command is received. Two commands are supported: Init, which starts a new flight cycle, and Read, which retrieves stored flight data from the external flash memory. When a new flight is requested, the system transitions to the INITIALIZATION_STATE, where all sensors and the flash memory are identified and configured using predefined parameters. Any unexpected response during this stage triggers a transition to the ERROR_STATE.

During nominal operation, the system enters the MISSION_EXECUTION state, in which all sensors are sampled at 12.5 Hz using interrupt-driven or data-ready mechanisms. Measurements from the sensing blocks are formatted into fixed 32-byte data packets and buffered before being written to external flash memory in page-sized blocks, optimizing throughput and memory usage.

Barometric measurements require the sequential acquisition of temperature and pressure samples to compute a compensated pressure value using the sensor’s internal calibration model. Although temperature is required for pressure compensation, it is not directly used by the flight-event detection logic. Consequently, when a state-transition event is detected, the temperature field of the corresponding data packet is overwritten with encoded event flags; otherwise, the temperature data are preserved.

Flight-state transitions are handled synchronously within the state machine using the deterministic event-detection algorithm described in [21]. The algorithm combines longitudinal acceleration and compensated barometric pressure to identify key flight phases. Launch is detected from a sustained increase in acceleration, while burnout is identified by a rapid decrease following the thrust phase. After propulsion ceases, apogee is determined when barometric pressure begins to increase monotonically, indicating loss of altitude and triggering the activation of the recovery system. Both single-stage and dual-stage deployment configurations are supported.

After landing, the user may request data retrieval through the serial interface. In the READ_STATE, the software sequentially reads all valid records stored in flash memory and transmits them in ASCII-encoded format. Upon completion, the system returns to the WAIT_STATE.

Although tailored to the present onboard computer, the linear and well-documented software architecture facilitates adaptation to other platforms. New sensors can be supported by adding dedicated drivers and extending the data structure, while modifications to the flight algorithm require changes only within the state-machine logic. This organization achieves a balance between clarity, reproducibility, and robustness, making the firmware suitable for both research and educational sounding-rocket platforms.

### 2.4 Overview of the HIL simulation

The dedicated hardware-in-the-loop simulation environment was developed to support validation of both the onboard computer hardware and its embedded software. Rather than acting as a standalone test stage, the HIL setup operates as an extension of the avionics architecture, allowing the flight microcontroller to execute using stimulus signals that reproduce the electrical, timing, and communication behavior of real sensors. This approach enables controlled, repeatable, and fully instrumented testing without requiring physical launches.

**Fig. 4:**
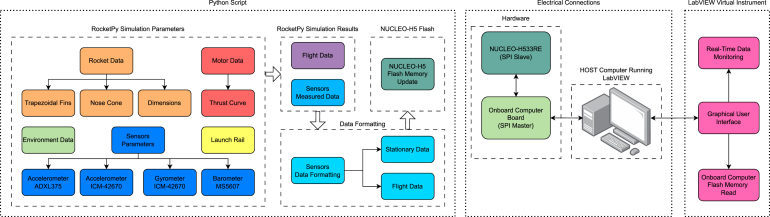
Block diagram of the HIL simulation.

Fig. 4 illustrates the overall structure of the HIL environment. Simulated flight trajectories are generated offline using a Python-based framework built around RocketPy [31], which provides six-degree-of-freedom (6-DOF) motion profiles based on vehicle, propulsion, and environmental parameters. From these trajectories, synthetic sensor measurements are constructed by incorporating sampling rate, noise, bias, and range characteristics derived from the sensors’ datasheets, enabling realistic representation of non-ideal sensing conditions. This approach provides flexibility across different vehicle configurations and mission scenarios, making it suitable for both educational activities and iterative research-oriented development of onboard avionics.

Because trajectory outputs are not directly compatible with embedded sensor interfaces, an intermediate formatting stage reproduces the behavior of each physical device, including register maps, SPI communication protocols, data-ready signaling, and timing constraints. Accurately reproducing these characteristics is essential to ensure that the onboard computer receives inputs that are indistinguishable from real sensors at the interface level.

The formatted datasets are stored in an external embedded platform acting as a sensor-emulation node. In the present implementation, a NUCLEO-H533RE board with an STM32H5 microcontroller operates as an SPI slave, serving the synthetic measurements to the onboard computer. Although this platform was selected due to availability and ecosystem compatibility, the HIL architecture is not tied to a specific device. Any embedded system capable of deterministic peripheral access and precise timing control can fulfill this role. Importantly, bare-metal execution (without an operating system) is essential to guarantee predictable timing behavior and reliable reproduction of sensor-level temporal characteristics.

During operation, the sensor-emulation node communicates with the onboard computer through the dedicated 20-pin HIL support connector, which exposes all sensor buses and associated control signals. This interface allows the HIL system to fully replace the physical sensing layer while preserving the original acquisition and processing chain. In this configuration, inertial (ADXL375 and ICM-42670-P) and barometric (MS5607) sensors are emulated with protocol-level fidelity, ensuring realistic system behavior.

Although the onboard magnetometer (MMC5983MA) is not currently included in the HIL simulation due to the absence of magnetic-field modeling in RocketPy, its integration is straightforward and is planned for future work related to magnetometer calibration and attitude-estimation algorithms [32].

A serial communication interface, available via USB CDC or external UART, enables real-time telemetry streaming to a host computer running a LabVIEW-based graphical user interface (GUI). During HIL operation, the onboard computer streams all acquired data to the GUI, enabling real-time visualization of acceleration, angular velocity, altitude, and internal state-machine transitions. The same interface is later used to retrieve the onboard log stored in flash memory for post-processing and verification.

Overall, the HIL environment enables end-to-end validation of the sensing, processing, and decision-making chain under realistic mission conditions. By externalizing sensor interfaces through a dedicated connector, HIL capability becomes an intrinsic feature of the avionics design rather than a temporary testing arrangement. Detailed operational procedures and workflow steps are presented in Section 6.1, forming the basis for the validation results and limitations discussed in Section 7.

## 3 Design files summary

All files needed to replicate and implement the data acquisition system designed in this work are available on Mendeley Data in an open-source file format, under the Creative Commons Attribution-NonCommercial 4.0 (CC BY-NC 4.0) license. Academic derivative works are permitted under this license, including adaptations such as modified components, integration of new sensors, or migration to alternative software frameworks, provided proper credit is given and the use remains non-commercial.

The hardware files were developed using Altium Designer Professional version 23.5.1, under an educational license. The .zip file corresponding to the Printed Circuit Board contains the schematics, PCB and Schematic libraries, PCB project, and all generated files used to manufacture the board, following the design constraints for JLCPCB (the chosen PCB manufacturer). In addition, a detailed bill of materials (BOM) for the PCB is also available, containing the specifications of all components used.

The software files for the project were developed using the STM32 Development Tools, including the Integrated Development Environment for STM32 (STM32CubeIDE) and STM32Cube initialization code generator (STM32CubeMX), in version 6.12.1. For the onboard computer, the project utilized the STM32CubeF4 firmware package version V1.28.2, while the HIL setup employed the STM32CubeH5 firmware package version V1.4.0. Both codes were compiled using the GNU Tools for STM32 toolchain (version 12.3.rel1). The codes were written using the HAL library and based on the documentation of the components used. In order to be user-friendly, the code includes brief comments regarding its main functionalities, already validated in field tests.

For the user interface, the Virtual Instrument files developed without commercial purpose using LabVIEW, under the Community Edition 2023 Q3, for controlling the HIL from a computer via serial communication, are included.

At last, experimental data obtained from the hardware-in-the-loop simulation of the developed onboard computer are available, including real time telemetry received through the serial interface, and the corresponding readings stored in its embedded flash memory. This dataset enables direct comparison between the simulated flight scenario and the hardware-acquired data, supporting independent validation of the system’s performance. Photos and videos of the rocket, the onboard computer, and the HIL setup used during the validation process are also available (see Table 1).

VECTOR_II_ONBOARD_COMPUTER_PCB.zip - Altium Designer Project for the onboard computer PCB.

**Table 1:** Onboard computer hardware design files.

Design filename	File type	Open source license	Location of the file
*VECTOR_II_ONBOARD_COMPUTER_PCB.zip*	*Altium Designer Project*	*CC BY NC 4.0*	https://doi.org/10.17632/dgf6yfv6p9.2.

*VECTOR_II_ONBOARD_COMPUTER_BOM.zip*	*BOM Spreadsheet*	*CC BY NC 4.0*	https://doi.org/10.17632/dgf6yfv6p9.2.

*VECTOR_II_ONBOARD_COMPUTER_SOFTWARE.zip*	*STM32 Project - Main Software*	*CC BY NC 4.0*	https://doi.org/10.17632/dgf6yfv6p9.2.

*VECTOR_II_ONBOARD_COMPUTER_HIL_SOFTWARE.zip*	*STM32 Project - HIL Software*	*CC BY NC 4.0*	https://doi.org/10.17632/dgf6yfv6p9.2.

*VECTOR_II_ONBOARD_COMPUTER_HIL_SAMPLE_DATA.zip*	*Sample Data*	*CC BY NC 4.0*	https://doi.org/10.17632/dgf6yfv6p9.2.

*VECTOR_II_ONBOARD_COMPUTER_MEDIA.zip*	*Onboard Computer Media*	*CC BY NC 4.0*	https://doi.org/10.17632/dgf6yfv6p9.2.

VECTOR_II_ONBOARD_COMPUTER_BOM.zip - Detailed Bill of Materials for the manufactured PCB.

VECTOR_II_ONBOARD_COMPUTER_SOFTWARE.zip - STM32CubeIDE project written in C for the developed onboard computer system.

VECTOR_II_ONBOARD_COMPUTER_HIL_SOFTWARE.zip – Collection of files used in the hardware-in-the-loop simulation setup. This archive includes:

(i) STM32CubeIDE project written in C for the developed hardware-in-the-loop simulation setup,
(ii) LabVIEW virtual instrument files for the Graphical User Interface develop to control HIL simulation, and
(iii) the Python Jupyter Notebook containing the initial RocketPy based synthetic flight data generation and formatting stage of the HIL workflow.

VECTOR_II_ONBOARD_COMPUTER_HIL_SAMPLE_DATA.zip – Data sets acquired during the hardware-in-the-loop simulation, including the real time telemetry received over the serial interface, the full flash memory logs extracted from the onboard computer, and a consolidated plot of the reconstructed flight profile.

VECTOR_II_ONBOARD_COMPUTER_MEDIA.zip - Compilation of photos and videos of the Vector II Project onboard computer hardware.

## 4 Bill of materials summary

Table 2 lists a partial BOM for replicating the onboard computer. Detailed information about all resistors and capacitors used is available on Mendeley Data at https://doi.org/10.17632/dgf6yfv6p9.2. The components are general-purpose electronic devices that can be sourced from various local and international suppliers.

**Table 2:** Onboard computer hardware bill of materials.

Designator	Component	Number	Cost per unit	Total cost	Source	Material type
PCB	Printed circuit board	5	$1.40 USD	$7.00 USD	https://jlcpcb.com/	Other

U11	STM32F407VGT6	1	$12.53 USD	$12.53 USD	https://www.digikey.com/en/products/detail/stmicroelectronics/STM32F407VGT6/2747117	Semiconductor

U1	ICM-42670-P	1	$3.66 USD	$3.66 USD	https://www.digikey.com/en/products/detail/tdk-invensense/ICM-42670-P/14319524	Semiconductor

U2	MS560702BA03-50	1	$8.72 USD	$8.72 USD	https://www.digikey.com/en/products/detail/te-connectivity-measurement-specialties/MS560702BA03-50/4700931	Semiconductor

U3	ADXL375BCCZ	1	$12.41 USD	$12.41 USD	https://www.digikey.com/en/products/detail/analog-devices-inc/ADXL375BCCZ/4376342	Semiconductor

U4	MMC5983MA	1	$2.79 USD	$2.79 USD	https://www.digikey.com/en/products/detail/memsic-inc/MMC5983MA/10452795	Semiconductor

U5	MAX3232IPWR	1	$1.00 USD	$1.00 USD	https://www.digikey.com/en/products/detail/texas-instruments/MAX3232IPWR/484752	Semiconductor

U6	AT45DB641E-SHN-B	1	$4.94 USD	$4.94 USD	https://www.digikey.com/en/products/detail/renesas-electronics-corporation/AT45DB641E-SHN-B/4901482	Semiconductor

U7	MIC29302WU-TR	1	$2.89 USD	$2.89 USD	https://www.digikey.com/en/products/detail/microchip-technology/MIC29302WU-TR/771594	Semiconductor

U8, U10	LP5907MFX-3.3/NOPB	2	$0.57 USD	$1.14 USD	https://www.digikey.com/en/products/detail/texas-instruments/LP5907MFX-3-3-NOPB/3906441	Semiconductor

U9	IRF7103TRPBF	1	$1.12 USD	$1.12 USD	https://www.digikey.com/en/products/detail/infineon-technologies/IRF7103TRPBF/811477	Semiconductor

Q1, Q2	MMBT6427-7-F	2	$0.21 USD	$0.42 USD	https://www.digikey.com/en/products/detail/diodes-incorporated/MMBT6427-7-F/717770	Semiconductor

Q3	BC817-40 RFG	1	$0.22 USD	$0.22 USD	https://www.digikey.com/en/products/detail/taiwan-semiconductor-corporation/BC817-40-RFG/7374204	Semiconductor

X1	ATS080BSM-1	1	$0.55 USD	$0.55 USD	https://www.digikey.com/en/products/detail/cts-frequency-controls/ATS080BSM-1/2292836	Other

Z1	PKLCS1212E4001-R1	1	$0.94 USD	$0.94 USD	https://www.digikey.com/en/products/detail/murata-electronics/PKLCS1212E4001-R1/1219314	Semiconductor

J_USB1	0473460001	1	$1.05 USD	$1.05 USD	https://www.digikey.com/en/products/detail/molex/0473460001/1782470	Semiconductor

D2	1N4007	1	$0.10 USD	$0.10 USD	https://www.digikey.com/en/products/detail/diotec-semiconductor/1N4007/18833652	Semiconductor

D4	PMEG4005CT,215	1	$0.26 USD	$0.26 USD	https://www.digikey.com/en/products/detail/nexperia-usa-inc/PMEG4005CT-215/2119874	Semiconductor

D3, D5 …D10	0603 SMD LEDs	6	$0.15 USD	$0.90 USD	https://www.digikey.com/en/products/filter/led-indication-discrete/105	Other

J_BAT_LOAD1, J_BAT_PWR1, J_LOAD1, J_LOAD2	2 × 1 Screw Conn	4	$0.81 USD	$3.24 USD	https://www.digikey.com/en/products/detail/phoenix-contact/1935776/2513905	Other

J_HIL1	10 × 2 Male Header	1	$0.23 USD	$0.23 USD	https://www.digikey.com/en/products/detail/adam-tech/PH2-20-UA/9830418	Other

J_COM	6 × 2 Male Header	1	$2.61 USD	$2.61 USD	https://www.digikey.com/en/products/detail/molex/0901303312/3303743	Other

JTAG_CONN1	5 × 2 Male Header	1	$0.19 USD	$0.19 USD	https://www.digikey.com/en/products/detail/adam-tech/PH2-10-UA/9830654	Other

P1	3 × 2 Male Header	1	$0.12 USD	$0.12 USD	https://www.digikey.com/en/products/detail/adam-tech/PH2-06-UA/9830396	Other

J_1	3 × 1 Male Header	1	$0.10 USD	$0.10 USD	https://www.digikey.com/en/products/detail/adam-tech/PH1-03-UA/9830289	Other

J_PWR_SW1, J_RST1	2 × 1 Male Header	1	$0.10 USD	$0.20 USD	https://www.digikey.com/en/products/detail/adam-tech/PH2-02-UA/9830267	Other

C6	T491S106K010AT	1	$1.65 USD	$1.65 USD	https://www.digikey.com/en/products/detail/kemet/T491S106K010AT/3724759	Other

C20, C21, C22, C29	T491C106K035AT	4	$0.99 USD	$3.96 USD	https://www.digikey.com/en/products/detail/kemet/T491C106K035AT/2336270	Other

C1 …C30	Various SMD Ceramic Capacitors	30	$0.1 USD	$3.0 USD	https://www.digikey.com/en/products/filter/ceramic-capacitors/60	Ceramic

R1 …R33	Various SMD Resistors	33	$0.1 USD	$3.3 USD	https://www.digikey.com/en/products/filter/chip-resistor-surface-mount/52	Ceramic

## 5 Build instructions

This section describes the construction and assembly of the onboard computer developed in this work, together with the supporting circuitry required for laboratory validation and hardware-in-the-loop operation. The focus is on the avionics hardware itself, including printed circuit board fabrication, power regulation, sensor interfacing, and pyrotechnic actuation circuits. Mechanical integration within a rocket airframe is addressed only insofar as it imposes dimensional, electrical, and robustness constraints on the avionics design.

All design files referenced in this section, including schematics, PCB layout, firmware repositories, and mechanical drawings, are provided in the Mendeley Data repository and summarized in the Design File Summary and Bill of Materials. The build instructions are organized to allow reproduction of the hardware both for bench-based HIL validation and for integration into small experimental rocket platforms. It is important to note that integration of the OBC into a flight-ready rocket platform inherently requires additional safety protocols and operational procedures that fall outside the scope of this article and are mandatory to experimental rocketry practice.

### 5.1 Printed circuit board fabrication and assembly

The OBC hardware is implemented on a four-layer printed circuit board designed to integrate sensing, processing, power management, data logging, and actuation functions within a compact footprint suitable for small experimental rockets. The PCB was fabricated by a commercial manufacturer, and its assembly was performed using a hybrid workflow: critical integrated circuits — including the MCU, inertial sensors, and flash memory — were mounted via pick-and-place followed by reflow soldering; passive components, connectors, and high-current terminals were soldered manually in a university laboratory. Fig. 5 presents a visual summary of the assembled hardware, including the division of functional areas and manufacturing process.

Although the prototype assembly was supported at no cost, a reference estimate based on the chosen PCB manufacturer (JLCPCB) indicates that the SMT assembly cost for a minimum batch of two boards with the same components would be approximately $52.61 USD, including setup fee ($25.37), stencil ($8.15), feeders loading ($15.20), SMT assembly ($0.63), and X-ray inspection ($3.26). Despite this, full manual assembly remains feasible, as the design avoids complex packages such as BGA components.Fig. 5Printed circuit board fabrication and assembly overview. (a) Top view of assembled PCB. (b) Bottom view of assembled PCB. (c) 3D model top view with functional areas. (d) Assembly process after reflow soldering.Fig. 5

**(a):**
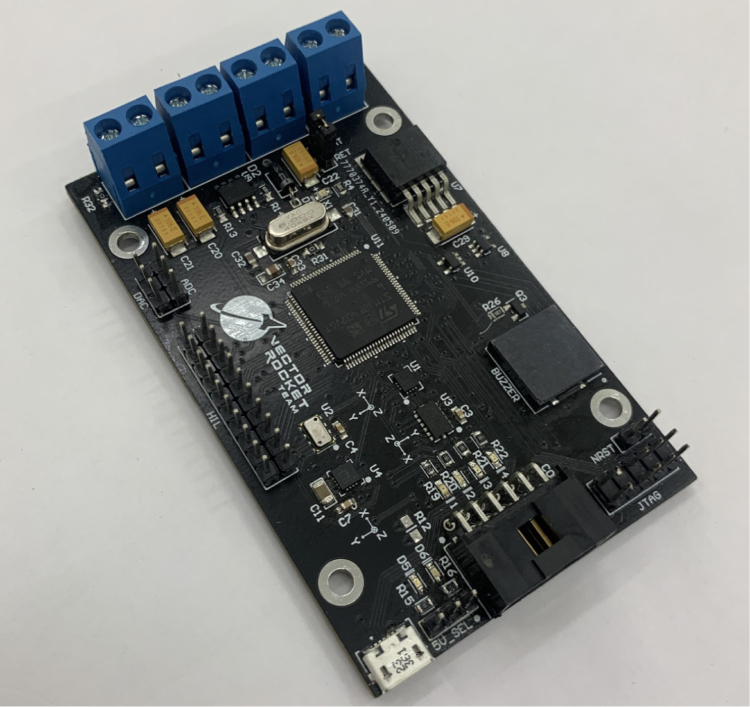

**(b):**
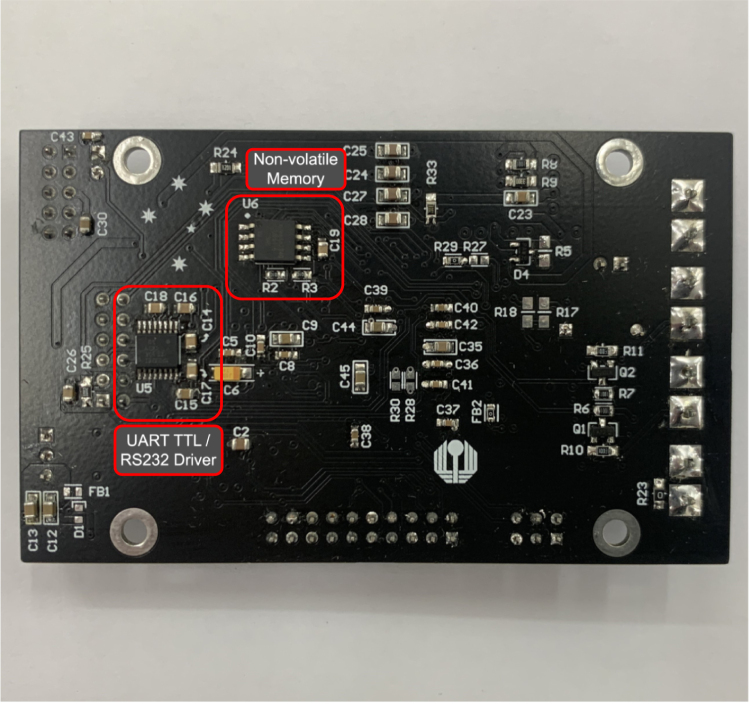

**(c):**
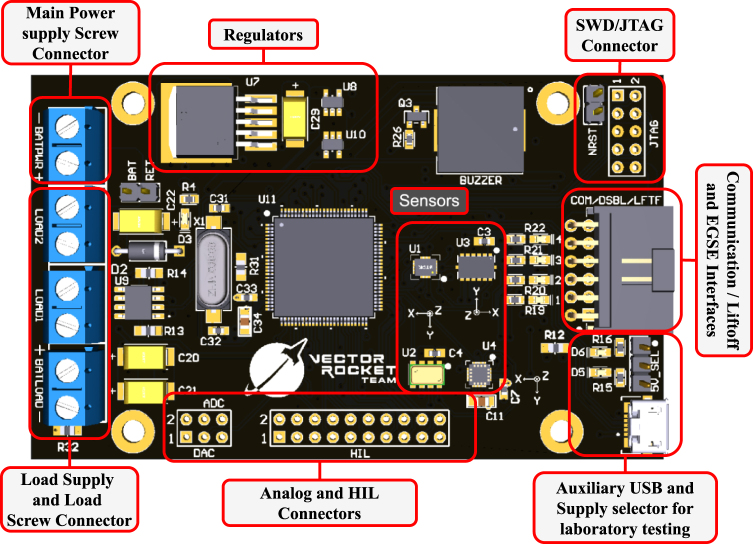

**(d):**
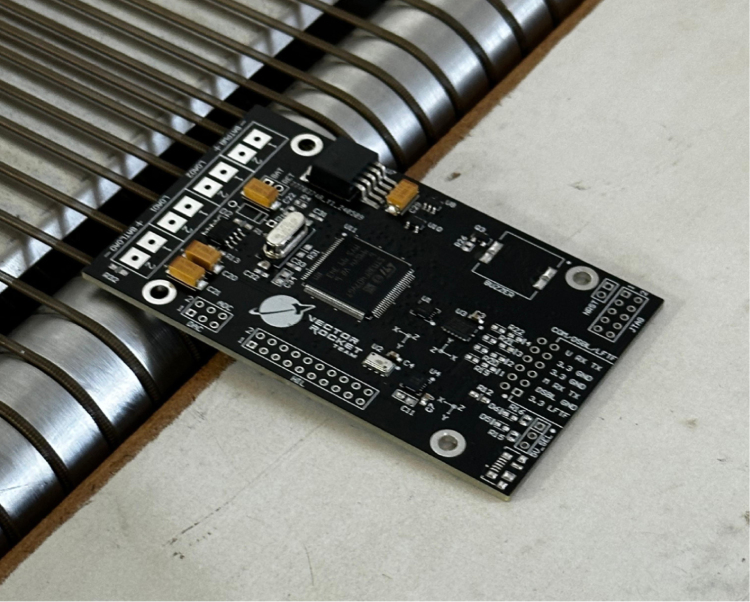

The stackup consists of signal layers on the top and bottom, with an internal ground plane on layer two and dedicated power distribution planes on layer three. This configuration supports controlled return paths, reduces electromagnetic interference, and provides low impedance supply rails for the sensors, microcontroller unit, and pyrotechnic driver circuits.

Component placement was arranged to clearly separate high-current switching loads from sensitive sensing and acquisition traces. As shown in Fig. 5(c), the left region of the board contains power input connectors, battery interfaces, and pyrotechnic actuation circuits, while digital sensors and the STM32F407VGT6 microcontroller are concentrated on the right side. This layout minimizes noise coupling into analog front-end stages and shortens critical SPI routing to the ADXL375, ICM-42670-P, MS5607, and MMC5983MA sensors. Decoupling capacitors and biasing networks were placed as close as possible to the respective devices, following manufacturer recommendations.

The PCB measures approximately 54.3 mm in width and 90 mm in length, including four M4 mounting holes positioned to interface with the internal support structure of the rocket. All connectors were placed at the board edges to facilitate stack-wise integration with the power subsystem and pyrotechnic loads, as well as to ease access during pre-launch flight checks. Screw terminal connectors were selected for the battery and pyrotechnic interfaces as a deliberate trade-off between mechanical robustness, accessibility, and cost. Given the short-duration nature of the mission and the constraints of academic platforms, this solution offers sufficient reliability without the added complexity and cost of aerospace-qualified connectors, while remaining widely adopted in similar electronics [17], [18], [19], [20].

Given these dimensions, and in the context of the Vector II case study discussed in Section 7, the available internal volume does not constrain the PCB footprint, and the current dimensions are compatible with the airframe. However, the architecture can be implemented in more compact form factors. Previous designs have achieved reduced dimensions (on the order of 70 mm × 15 mm) by using smaller component packages and higher integration density [33]. This approach increases assembly complexity, cost and reduces suitability for manual soldering, and was therefore not adopted in this work.

Beyond mechanical form factor considerations, the routing strategy emphasizes controlled power distribution, thermal robustness, and separation of analog and digital domains. Power delivery networks are reinforced with widened traces and localized copper pours that improve heat spreading around the five volt regulator, supported by thermal vias to the internal planes. Sensitive analog sections are isolated shielding copper areas tied to ground through 0 Ω jumpers that allow reconfiguration and reduce coupling from high-speed digital lines. Overall, the routing strategy prioritizes clear return paths and mechanical reliability under launch vibration conditions.

### 5.2 Power regulation and distribution

As introduced in Section 2, the system employs two separate power domains, each supplied by its own Li-ion battery pack (nominal 7.4V): one dedicated to the avionics electronics and another to the pyrotechnic loads. While the two domains use independent power sources, they share a common electrical ground reference to ensure proper signal integrity and reliable actuation. This architecture allows high-current pyrotechnic actuation without inducing voltage drops or transients in the avionics supply.

In the avionics domain, the board supports multiple power input paths to facilitate flight operation, bench testing, and hardware-in-the-loop workflows. The primary supply can be provided through the dedicated battery connector, the USB port, or the debug interface.

**Fig. 6:**
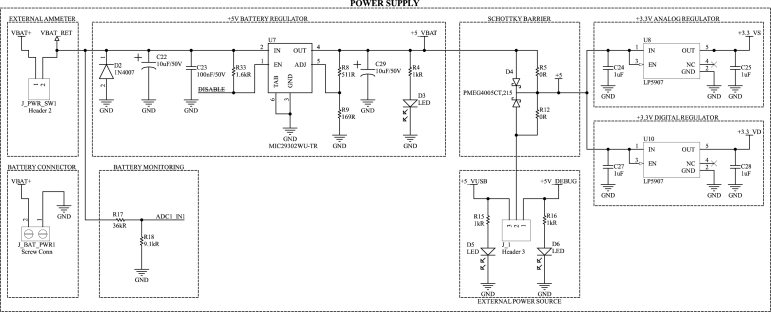
Power supply schematic, showing regulator hierarchy, battery paths, and source selection.

As shown in the simplified power path of Fig. 6, the battery input first passes through the in-line header J_PWR_SW1, which allows current measurement using an external ammeter during qualification tests. Removing the jumper electrically isolates the system, allowing measurement of battery consumption without modifying the wiring harness. Importantly, for a flight scenario, the battery must be powered by the battery connector and J_PWR_SW1 short-circuited, as the jumper is not recommended for flight. A reverse-protection diode (1N4007) is connected across the battery terminals to clamp polarity inversion events and prevent damage during testing or harness connection.

After the measurement stage, the battery drives a high-current MIC29302WU linear regulator [29], which generates the main +5V rail used by digital circuitry, communication interfaces, and downstream low-noise regulators. The output voltage (+5_VBAT in the schematic) is configured through $R_{8}$ and $R_{9}$ resistive divider:

(1) $$R_{8}=R_{9}\left(\frac{V_{\mathrm{OUT}}}{1.240}-1\right).$$

For $V_{\mathrm{OUT}}=5\;V$, the resistor ratio is $\frac{R_{8}}{R_{9}}=3.032$, and resistors values must be chosen to ensure regulation under no load condition accordingly to the device’s specification in the datasheet.

The regulated +5V output is then routed to a PMEG4005CT215 Schottky barrier, which isolates the regulated battery supply from external power sources such as USB or the JTAG interface, as illustrated in Fig. 6. This configuration preserves the ability to power the board either from the flight battery or from bench supplies while preventing reverse current flow into the regulator or the battery. If a forced single-source configuration is required, the Schottky stage may be bypassed by populating the optional 0Ω resistors R5 and R12, disabling automatic source selection.

Exclusively for HIL simulation or grounding tests, power input can alternatively be supplied through the DEBUG or USB connectors. The selector header J_1 chooses which external source powers the board: the upper position routes power from the JTAG connector, while the lower position selects USB power. The USB input includes a transient suppression TVS diode for surge and ESD protection; however, no equivalent protection device is present on the debug power path, requiring caution when applying external power through this connector. This arrangement enables programming, firmware debugging, and bench testing without installing the flight batteries.

Downstream from the regulated +5V rail, two independent LP5907 low-noise regulators [30] generate +3.3V supplies: one for digital logic and sensor interfaces, and another reserved for the analog power domains of the inertial sensors.

The use of low-noise LDO regulators with high power-supply rejection ratio (PSRR) ensures stable sensor operation while minimizing supply-noise coupling. Although the system is primarily designed for direct operation from a two-cell (2s) Li-ion battery, this regulation architecture also supports integration with more complex power-distribution systems, including those based on switching regulators. In such configurations, the LDO stages inherently attenuate switching noise, allowing the onboard computer to maintain reliable operation without requiring hardware modifications.

To quantify the power demand of the avionics subsystem, a consumption estimate was derived based on nominal values obtained from the component datasheets under the configured operating conditions. The results are summarized in Table 3. The sensor contribution corresponds to an aggregated estimate based on their respective operating modes, while the microcontroller and memory values represent realistic values for the implemented configuration. All functional loads are supplied from the +3.3V rail, implying that the same current flows through both the primary and secondary regulation stages. The additional current associated with the MIC29302WU quiescent consumption is approximately 7mA and has to be accounted in the power dissipation analysis.

Considering a 2S Li-ion battery input (nominal 7.4V, up to ≈8.4V when fully charged), adopted here as the nominal operating condition for this estimation, the power dissipation across the linear regulators can be estimated as:

(2) $$P_{loss,5V}=\left(V_{in}-5\right)\cdot I_{5V}\approx \left(8.4-5\right)\cdot 0.069\approx 0.24\;W,$$

(3) $$P_{loss,3.3V}=\left(5-3.3\right)\cdot I_{3.3V}\approx \left(5-3.3\right)\cdot 0.062\approx 0.11\;W,$$

where $V_{in}$ is the battery input voltage, $I_{3.3V}$ is the total load current drawn from the +3.3V rail, and $I_{5V}$ includes both the load (62mA) and the regulator quiescent (7mA) currents consumption. The terms $\left(V_{in}-5\right)$ and (5−3.3) represent the voltage drops across the primary (MIC29302WU) and secondary (LP5907) LDO regulators, respectively.

**Table 3:** Datasheet-based power consumption of the onboard computer under nominal operating conditions.

Component	Current
	(mA)
STM32F407VGT6 (100 MHz est.)	50
Sensors (IMU, accelerometer, barometer, magnetometer)	2.5
AT45DB641E (avg.)	5
MAX3232	0.3
LEDs (4x)	4

**Total load (3.3 V rail)**	≈62 mA

The regulator supplying the analog domain draws a negligible current and does not significantly contribute to the overall power dissipation. Therefore, the total power dissipation in the regulation stages remains below 0.35W under realistic operating conditions. This low power level, combined with the short mission duration and the use of copper planes for heat spreading, ensures adequate thermal performance.

Battery voltage is monitored through a resistive divider connected to one of the microcontroller ADC channels:

(4) $$V_{\mathrm{ADC}}=V_{\mathrm{BAT}}\cdot \frac{R_{18}}{R_{18}+R_{19}}$$

where $R_{18}$ and $R_{19}$ are the resistive divider resistors that set the ADC input voltage (in ohms), $V_{\mathrm{BAT}}$ is the battery voltage (in volts), and $V_{\mathrm{ADC}}$ is the resulting voltage applied to the ADC input. With the implemented resistor values, the ratio becomes:

(5) $$V_{\mathrm{ADC}}=0.2018\cdot V_{\mathrm{BAT}}$$

Given the ADC full-scale input of 3.3V, the maximum measurable battery voltage without the risk of damage is 16.35V.

### 5.3 Pyrotechnic actuation circuits

**Fig. 7:**
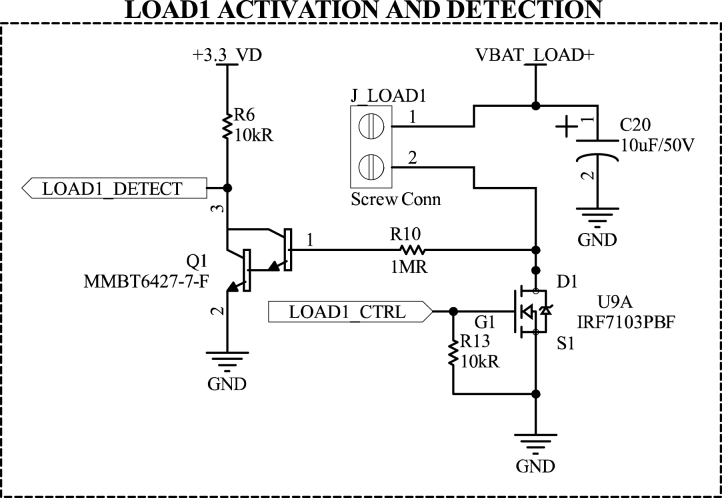
Pyrotechnic actuation and continuity-monitoring circuit for a single channel.

Fig. 7 shows the schematic of the pyrotechnic actuation circuit. The system features two identical channels; however, only one is depicted for clarity. In the current implementation, the two channels may operate redundantly or trigger independent deployment events, depending on the recovery configuration.

Each channel integrates solid-state actuation, continuity monitoring, and a dedicated power rail to ensure reliable ignition during high-acceleration flight. The pyrotechnic load connects to a screw terminal whose positive pin is driven by an independent battery line decoupled with a 10μF, 50V tantalum capacitor placed near the connector to reduce source impedance and support peak discharge current. The return path is routed through the MOSFET drain, while the source is tied to the common ground shared between avionics and deployment subsystems. During activation, the microcontroller drives the MOSFET gate high, allowing current from the dedicated rail to flow to the load; a 10kΩ gate-to-source resistor accelerates discharge and prevents unintended turn-on due to noise or floating logic states.

Continuity monitoring is performed through a high-impedance sensing network that biases the MOSFET drain through a 1MΩ resistor into the base of an MMBT6427-7-F Darlington transistor. The transistor emitter is grounded, and the collector is pulled up to the +3.3V logic domain through a 10kΩ resistor, providing a digital LOAD_DETECT signal to the microcontroller. When a pyrotechnic or emulated load is present, a small leakage current enables conduction in the Darlington stage, pulling the logic node low; otherwise, the node remains high due to the pull-up resistor. Because the sensing network is upstream of the MOSFET, continuity remains measurable without heating the igniter and does not interfere with actuation.

A MOSFET-based switching stage using the IRF7103PBF is adopted instead of electromechanical relays due to its superior reliability under vibration. Mechanical relays are susceptible to intermittent conduction, conduction failures, and contact bounce, which can occur during ascent and high-vibration environments [34]. Since ignition is a single-shot, mission-critical event, these failure modes are unacceptable. The MOSFET approach, or solid-state relay solutions, eliminates mechanical contacts, provides deterministic switching, and offers lower on-resistance while maintaining robustness to high-G launch conditions.

In addition to flight operation, the pyrotechnic actuation circuitry was explicitly designed to support safe ground testing and HIL validation. During laboratory operation, the output terminals can be connected to passive resistive loads or emulated igniters, allowing verification of continuity monitoring, command logic, and actuation timing without introducing thermal or mechanical hazards associated with live pyrotechnic devices.

Beyond purely emulated loads, the same PCB and HIL framework can be used to validate real deployment hardware intended for flight — such as igniters, squibs, or release mechanisms — under controlled pre-launch conditions [35], [36]. This approach allows critical subsystems to be exercised under representative conditions while maintaining a controlled and repeatable laboratory environment, significantly reducing risk during subsequent launch operations.

## 6 Operation instructions

This section is divided into three main parts. First, it presents the operation of the hardware in a laboratory environment using HIL simulation, from data generation with RocketPy to real-time execution. Next, although a real flight is outside the scope of this work, guidelines for OBC usage in a flight scenario are provided. Finally, post-flight data handling is discussed for both HIL and real-flight cases.

### 6.1 Hardware-in-the-loop simulation operation

This subsection presents a laboratory workflow for operating the HIL simulation environment introduced in Section 2.4, enabling systematic and repeatable validation of the OBC and associated subsystems without requiring a physical launch.

**Fig. 8:**
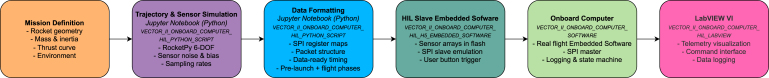
Overview of the hardware-in-the-loop (HIL) workflow adopted in this work, from mission definition to real-time execution on the onboard computer. Each nomenclature in italic identifies the element within the dataset repository.

Fig. 8 summarizes the HIL workflow adopted in this work, from mission definition to real-time execution on the onboard computer. Each block corresponds to scripts, firmware, or software modules available in the Mendeley Data repository, enabling full reproduction of the validation pipeline.

Although RocketPy is employed in the present implementation as the 6-DOF trajectory generator, the proposed HIL framework is not tied to a specific simulation tool. Any flight-dynamics model capable of providing time-resolved kinematic and environmental data can be integrated, provided that the output trajectories are mapped to the expected sensor interfaces.

For clarity, the workflow is organized into four stages: (i) mission and trajectory definition, (ii) sensor-level data synthesis and formatting, (iii) embedded HIL slave execution, and (iv) real-time execution of the flight software on the onboard computer with telemetry and logging. This separation simplifies debugging, facilitates incremental validation, and allows individual components to be modified without affecting the overall process.

The following subsections describe each stage with emphasis on the steps required to reproduce the setup using the provided scripts, firmware, and design files.

#### 6.1.1 Rocketpy and preparation of simulation data

The HIL workflow begins with the execution of the VECTOR_II_ONBOARD_COMPUTER_HIL_PYTHON _SCRIPT Jupyter Notebook, which generates the synthetic flight dataset. The script uses RocketPy to compute a six-degree-of-freedom trajectory based on:

• Environmental conditions: gravity, latitude, longitude and elevation (see block 2 of the script);
• Motor definitions, including thrust curve, grain characteristics, burn time, and nozzle geometry (block 3);
• Rocket definitions: Rocket geometry, mass distribution, aerodynamic model (block 4);

The dataset and validation results presented in this work are based on the Vector II rocket parameters, described in Section 7, ensuring that the HIL tests are consistent with a realistic university rocket design. RocketPy also provides visualization and summary tools for the defined vehicle (block 7).

Once the trajectory is computed (block 8), the notebook generates synthetic sensor measurements using RocketPy’s built-in sensor modeling routines (block 9). Sensor parameters, defined in block 5 and derived from the respective datasheets, are used to apply full-scale range, noise density, sampling rate, and bias characteristics. This results in a time-aligned dataset with realistic measurement variability. Additional stationary segments representing pre-launch conditions are generated to support system initialization and bias estimation.

For reproducibility, the synthetic sensor data were generated using a locally bundled version of RocketPy included in the Mendeley Data repository. Minor adjustments were applied to the internal gravitational handling of the accelerometer model to ensure consistency with the adopted inertial reference convention, guaranteeing that stationary conditions yield the expected 1 g vertical acceleration.

Further details on RocketPy are available in the library documentation [31].

#### 6.1.2 Data formatting and embedded software flashing

The raw trajectory outputs generated by RocketPy are not directly compatible with the HIL simulation. Using the Jupyter Notebook script VECTOR_II_ONBOARD_COMPUTER_HIL_PYTHON_SCRIPT (blocks 9 and 10), the synthetic sensor measurements are formatted to match the behavior of the physical sensors used onboard. This includes reproducing register maps, SPI command–response structure, data-ready signaling, chip-select timing, and transitions between stationary (pre-launch) and dynamic (in-flight) phases.

This formatting step is essential to guarantee that the OBC cannot distinguish between simulated and real sensors at the interface and protocol levels.

Once formatted, block 11 updates the .c source files where the datasets are stored. These files are then compiled into the embedded firmware of the HIL slave hardware using STM32CubeIDE. In the current implementation, the datasets are stored as arrays in the flash memory of a NUCLEO-H533RE development board configured as an SPI slave.

Any modification to sensor models, sampling rates, or register mappings requires regenerating the datasets and recompiling the HIL slave firmware, followed by flashing the updated binary using STM32CubeIDE.

#### 6.1.3 Bench setup and electrical connections

With the HIL slave firmware deployed, the next step consists of assembling the laboratory bench setup that electrically connects the onboard computer to the sensor-emulation hardware. This setup is intentionally simple and relies on standard development tools to facilitate reproducibility in academic laboratories.

As described in Section 2.4, the current implementation emulates the inertial (ADXL375 and ICM-42670) and barometric (MS5607) sensors. The inertial sensors share the same SPI bus in the flight hardware. Consequently, the setup requires SPI1 and SPI2, three chip-select signals, and one interrupt line associated with the ADXL375 sensor, all routed through the HIL support connector.

Fig. 9(a) depicts the HIL support connector on the onboard computer, while Fig. 9(b) shows the corresponding pin-to-pin wiring between the onboard computer and the NUCLEO-H533RE HIL slave board. Although the present implementation uses an STMicroelectronics NUCLEO platform, the HIL architecture is not restricted to a specific device. Any external platform capable of implementing the required SPI slave protocols and deterministic timing behavior can be adapted to this interface.

Electrical connections may be implemented using individual jumper wires for exploratory laboratory work, given the 2.54 mm pin pitch. For repeated experiments or teaching laboratories, keyed ribbon cables are recommended to improve robustness and reduce wiring errors. Both systems must share a common electrical ground reference.Fig. 9HIL support connector. (a) Schematic view of the connector on the onboard computer. (b) Wiring diagram showing the electrical connections between the onboard computer and the NUCLEO-H533RE used for HIL sensor emulation.Fig. 9

**(a):**
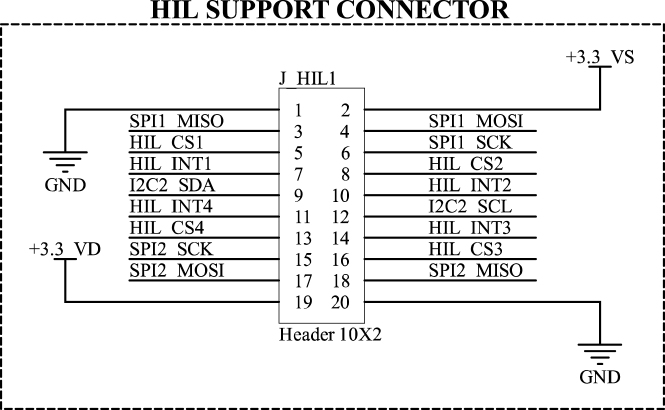
.

**(b):**
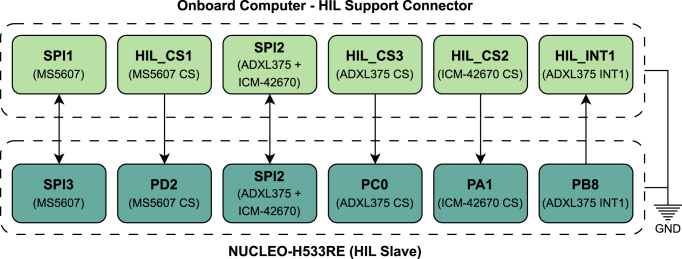
.

During HIL operation, the setup is typically powered using laboratory bench supplies, eliminating the need for flight batteries. Alternatively, power may be supplied through the USB or DEBUG connectors of the onboard computer, as described in Section 5.2. The selector header J_1 defines the active power source. When powering the system through the DEBUG connector, particular care must be taken, as this path does not include transient or ESD protection.

During the simulation, the OBC acts as the SPI master, while the NUCLEO-H533RE provides protocol-accurate sensor responses stored in its flash memory. The OBC executes the embedded firmware (VECTOR_II_ ONBOARD_COMPUTER_SOFTWARE), with the chip-select signals redefined from the physical sensor pins to the corresponding pins of the HIL support connector. In parallel, the NUCLEO-H533RE runs the HIL slave firmware (VECTOR_II_ONBOARD_COMPUTER_HIL_H5_EMBEDDED_SOFTWARE), which streams the synthetic sensor data in response to SPI transactions.

**Fig. 10:**
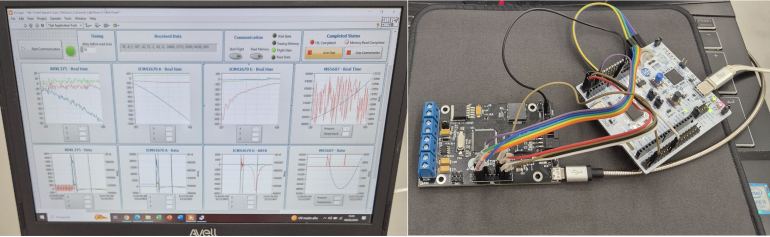
Image of the setup during a test. LabVIEW UI graphs after the HIL simulation and OBC to the NUCLEO-H533RE board wiring connection in Fig. 9(b).

A serial communication interface (USB CDC or UART) links the onboard computer to a host PC running the LabVIEW-based graphical interface, which provides real-time telemetry and state visualization. Fig. 10 shows the OBC, the HIL slave board, and the LabVIEW VI during a test session, where sensor data are visualized in real time. Next subsection explains the LabVIEW operation, the data analysis and graphs are left for the validation section.

Detailed instructions for running the HIL operation are provided in the following subsection, while data retrieval is described later in Section 6.3.

#### 6.1.4 Running the simulation and monitoring telemetry

After completing the electrical setup, the user can launch the LabVIEW Virtual Instrument (VI), which serves as the primary human–machine interface in the HIL implementation. The VI provides real-time visualization of emulated sensor measurements, state-machine transitions, and logging status. It also allows the operator to trigger the flight simulation and retrieve stored data. The front panel includes controls for logging configuration, communication management, and visualization of sensor streams from the ADXL375, ICM42670, and MS5607 devices.

**Fig. 11:**
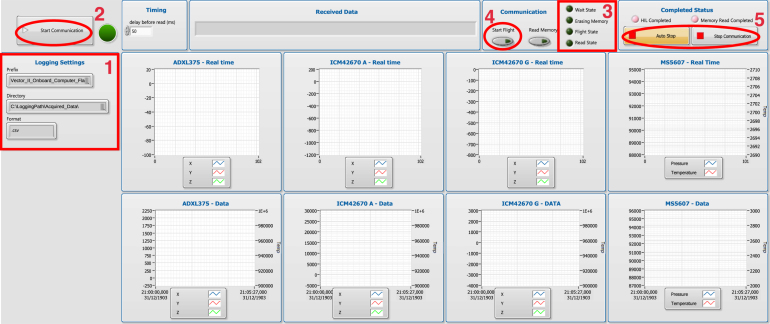
LabVIEW Virtual Instrument front panel GUI.

Fig. 11 highlights the main interface elements, including communication controls, status indicators, real-time plots, and post-acquisition summary graphs.

The recommended procedure for running an HIL simulation using the LabVIEW VI is as follows:

1. Connect the NUCLEO-H533RE to the onboard computer through the 20-pin HIL connector, ensuring a common ground reference between boards;
2. Provide power using a laboratory bench supply or through external connectors as described in Section 5.2, configuring the J_1 selector header accordingly;
3. Connect the onboard computer to the host PC through the serial communication interface (USB CDC virtual COM port or external UART) to enable telemetry, logging, and command transmission;
4. Launch the LabVIEW VI and configure logging parameters (directory, filename prefix, and output format) using the controls highlighted in Area 1 of Fig. 11;
5. Press the *Start Communication* button (Area 2) to establish the serial link;
6. Verify the status indicators in Area 3. The system initially remains in the *WAIT* state. Press the *Start Flight* button (Area 4) to command the onboard computer to begin a new flight session;
7. Upon receiving the command, the *Erasing Memory* indicator remains active for approximately 220 s while the onboard flash memory is cleared. During this period, the red LED on the onboard computer blinks once and remains off until completion;
8. After memory erasure, the system enters the *READY* state, indicated by the *Flight State* indicator in Area 3 and a slow blinking green LED on the onboard computer;
9. At this stage, monitor the real-time sensor data in the upper plots and confirm that packet fields are consistent with the expected stationary condition;
10. When ready to initiate the simulated flight, press the *User Button* on the NUCLEO-H533RE to begin streaming the synthetic flight dataset stored in its flash memory;
11. Monitor the real-time plots as the simulation progresses through the trajectory, showing acceleration, angular-rate, and altitude profiles;
12. Allow the simulation to run to completion. If *Auto Stop* mode (Area 5) is enabled, the VI automatically terminates communication after the final packet. Otherwise, press *Stop Communication* to manually terminate logging and close the communication channel;
13. After completion, summary plots in the lower portion of the interface display the accumulated sensor data and flight state transitions for post-acquisition inspection;
14. Retrieve the logged data files from the configured directory for post-processing and analysis.

#### 6.1.5 Automated execution and future extensions

A fully automated HIL workflow can be achieved by replacing the LabVIEW VI with a Python-based interface or script. In this configuration, trajectory generation, firmware flashing, dataset loading, command sequencing, and telemetry acquisition are executed programmatically, enabling unattended operation and large-scale Monte Carlo simulation campaigns.

The automation pipeline also enables systematic variation of environmental parameters (e.g., atmospheric density, wind profiles, and thrust curves), allowing evaluation of the robustness of onboard state-estimation and event-detection algorithms across a wide range of flight conditions.

The following considerations are recommended when implementing an automated workflow:

• **Safety considerations**: Disable pyrotechnic outputs in the embedded software or route their terminals to passive resistive loads during HIL operation. Hardware-level emulation of these channels may be incorporated for testing, as discussed in the following section.
• **Closed loop actuation testing**: Although not applicable to a solid-propellant vehicle considered in the Vector II project, the same HIL framework can be extended to support closed-loop testing of propulsion or valve control subsystems in future liquid or hybrid rocket platforms.

### 6.2 Configuration and wiring of the avionics for a real flight

The transition from laboratory validation using the HIL setup to real-flight operation does not require modifications to the flight logic or state-machine implementation of the onboard computer. The embedded software is structured using hardware-abstraction layers; therefore, replacing the HIL sensor interface with the physical onboard sensors primarily involves reassigning SPI chip-select and interrupt mappings to the corresponding hardware pins. Acquisition loops, filtering routines, event-detection logic, and logging procedures remain unchanged, preserving the behavior validated under HIL conditions.

Although the software logic remains identical, real-flight operation requires careful configuration of physical interfaces, power domains, and safety procedures. In particular, the pyrotechnic subsystem must be validated through staged ground tests before integration into the vehicle.

Prior to installing live charges, the following checks are recommended:

• Verify sensor communication through their dedicated SPI interfaces;
• Confirm correct lift-off signal detection using a simulated break-wire or rail-separation mechanism;
• Test pyrotechnic outputs using resistive loads or inert emulators to validate continuity monitoring, command sequencing, and activation timing;
• Perform a complete telemetry and logging check while the vehicle is mechanically assembled but with ignition and deployment channels electrically inhibited.

Only after these steps should live deployment charges be installed and verified under controlled outdoor conditions, following institutional and local safety regulations. A complete launch and range-safety protocol is beyond the scope of this work^1^; the procedure below focuses exclusively on the configuration and operational sequence of the onboard computer.

The recommended operational sequence for preparing the OBC for flight is:

1. Flash the flight firmware onto the microcontroller, ensuring that all mission-specific parameters and #define configuration options (e.g., single-stage or dual-stage recovery) are correctly set;
2. Install the avionics battery (main electronics supply). The pyrotechnic battery should remain disconnected at this stage;
3. Install the lift-off detection mechanism (e.g., break-wire or removable shorting plug) and verify correct detection behavior;
4. Connect the pyrotechnic charges to their designated terminals. Two recovery configurations are supported:
  • Redundant mode: both charges fire simultaneously at apogee;
  • Dual-stage mode: a drogue parachute is deployed at apogee and a main parachute at a predefined altitude (configured at compile time).
5. Connect the onboard computer to a laptop, preferably via the UART/RS-232 interface. Verify communication using LabVIEW or a serial terminal and place the system in flight-ready mode;
6. Confirm that the system transitions to the *READY* state and that all sensor data are being acquired nominally;
7. Connect the pyrotechnic battery only after the vehicle is positioned on the launch rail and all personnel are at a safe distance.

**Fig. 12:**
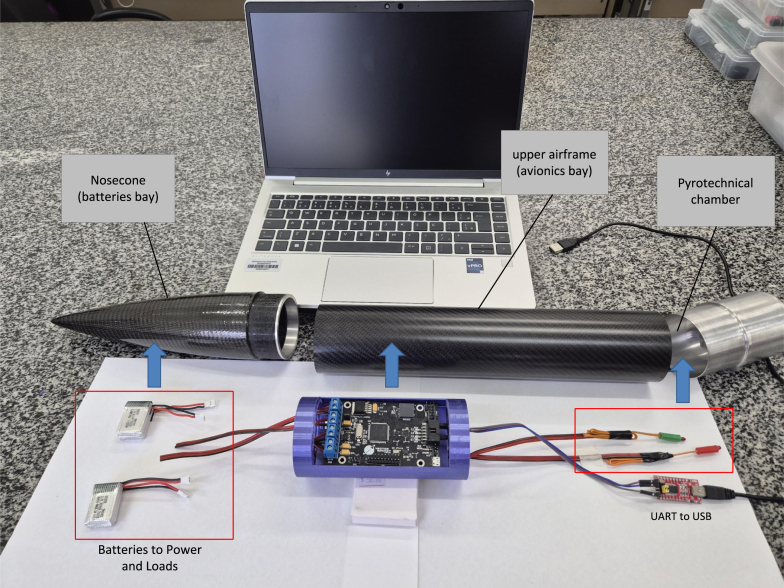
Representative wiring configuration of the onboard computer during flight preparation.

Fig. 12 illustrates a representative wiring configuration for flight preparation. For clarity, the components are shown outside the rocket body.

### 6.3 Post flight data retrieval and analysis

Following either a real flight or a bench simulation using the HIL setup, the onboard computer stores the complete sensor timeline in its non-volatile flash memory. The data-extraction procedure is identical in both cases and is preferably performed using the LabVIEW VI, which logs the decoded telemetry stream to selected directories for subsequent processing. Once retrieved, the dataset can be compared with pre-flight 6-DOF simulations or HIL-generated reference trajectories. Consequently, it enables quantitative assessment of model fidelity, algorithm robustness, and environmental effects such as aerodynamic drag, wind conditions, and sensor noise.

The recommended workflow for retrieving and inspecting flight data is:

1. Connect the onboard computer to the host PC via the serial interface (USB CDC or UART);
2. Launch the LabVIEW VI and configure logging parameters (directory, filename prefix, and output format) using the controls highlighted in Area 1 of Fig. 11;
3. Press the *Start Communication* button (Area 2) to establish the serial link. The system initially reports the *WAIT* state;
4. Issue the memory-extraction command by clicking *Read Memory* (Area 4). The *READ* state indicator (Area 3) confirms that the flash readout routine has started;
5. Monitor the real-time plots as the data stream is decoded. Acceleration, angular-rate, and altitude profiles are displayed in the upper plots. During this process, the red LED on the onboard computer blinks slowly until completion;
6. Allow the extraction process to complete. If *Auto Stop* (Area 5) is enabled, the VI terminates automatically after the final packet. Otherwise, stop the session manually using the *Stop Communication* button;
7. Inspect the summary plots in the lower region of the interface, which aggregate sensor data and state transitions for rapid post-acquisition review;
8. Retrieve the logged files from the configured directory for detailed analysis.

In this configuration, the LabVIEW VI acts as a graphical interface and logging utility, storing the received data in .CSV format for straightforward analysis with standard tools. The communication protocol, however, remains fully accessible through generic serial interfaces, ensuring that data retrieval is not dependent on proprietary software.

Therefore, if the LabVIEW VI is unavailable, data can be retrieved using any standard serial terminal via either the UART interface or the USB virtual COM port. After power-on or reset, the OBC remains in the *WAIT* state. Sending the ASCII command Read transitions the system to the *READ* state and initiates sequential transmission of the flash-memory contents as comma-separated data packets. The stream continues until all valid memory locations are read, followed by a completion message, after which the system returns to the *WAIT* state.

For post-flight handling, ensure that pyrotechnic outputs are disconnected or routed to passive loads, as the memory-extraction routine does not require any actuation hardware. The serial communication link should not be interrupted during flash readout, as incomplete transfers may corrupt the retrieved data. Ensure that the onboard battery has sufficient charge for the entire extraction process, or replace it with a regulated bench supply matching the nominal system voltage.

## 7 Validation and characterization

The validation and characterization of the proposed onboard computer hardware and software were carried out using the hardware-in-the-loop framework described in Sections 2.4, 6.1. Instead of relying on launch campaigns, which are costly, logistically complex, and inherently risky, the adopted approach enables controlled, repeatable, and parameterized evaluation of system performance. The validation focused on system-level metrics, including timing determinism, data integrity, and robustness under variations in mission conditions. Event-detection performance was assessed quantitatively in terms of detection delay relative to simulated ground truth.

A representative mission scenario derived from the ongoing Vector II university rocket project was adopted to define realistic flight dynamics, sensor excitation levels, and mission timelines. In this context, the Vector II vehicle serves as a case study to generate physically consistent trajectories for evaluation, without constraining the applicability of the proposed architecture to a specific platform.

The Vector II case study corresponds to a small experimental solid sounding rocket with an overall length of approximately 1100 mm and a body diameter of 65 mm (excluding aerodynamic fins). Its internal structure follows that shown in Fig. 1 and accommodates the battery module, onboard computer, recovery system, and solid-propellant motor. The propulsion system is based on a sucrose–sodium nitrate propellant formulation, cast as an unconstrained hollow cylindrical grain and housed in an aluminum combustion chamber with a stainless-steel De Laval nozzle. The motor contains approximately 300 g of propellant and produces an average thrust of about 215 N over a burn duration of roughly 1.7 s. Based on the measured thrust curve obtained in the project [37], [38], a total impulse around 365 N s, and assuming a lift-off mass around 2.00 kg, nominal simulations from RocketPy predict an apogee between 750 and 850 m under standard atmospheric conditions. These parameters were used exclusively to generate physically consistent trajectories for the HIL validation.

Table 4, Table 5 summarize the parameters used as inputs to the RocketPy script described in Section 6.1.1. Using these inputs, the script generates the simulated flight campaign, producing synthetic acceleration, angular velocity, and pressure-derived altitude signals from the 6-DOF vehicle model implemented in RocketPy.

**Fig. 13:**
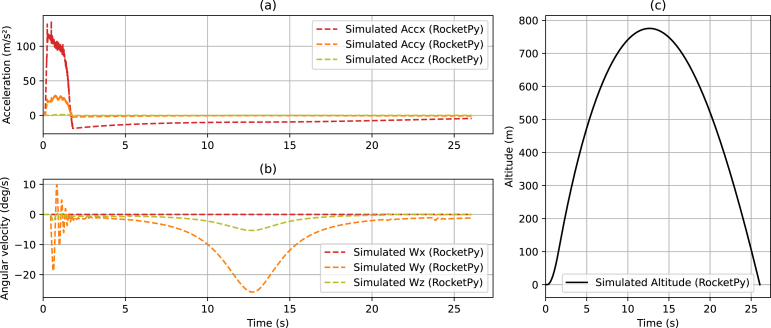
Simulated inertial flight data generated by RocketPy. (a) Acceleration. (b) Angular velocity. (c) Altitude.

The resulting flight data are shown in Fig. 13, and all datasets are provided in the repository. These signals correspond to the ground-truth trajectory generated by RocketPy, expressed in an inertial reference frame. In this representation, acceleration does not include the gravitational component (i.e., it corresponds to the vehicle’s kinematic acceleration), and altitude is defined relative to the launch point, starting from zero at lift-off.

Synthetic measurements are generated from the trajectories shown in Fig. 13 using sensor models configured according to the characteristics listed in Table 6, extracted from the respective datasheets.

**Table 4:** Environment and launch conditions used in RocketPy simulation.

Parameter	Value	Unit
Latitude	−23.20	deg
Longitude	−45.87	deg
Elevation	10	m
Launch rail length	5.2	m
Launch inclination	85	deg
Launch heading	0	deg

**Table 5:** Rocket and solid motor physical parameters used in RocketPy.

Solid motor		Rocket body	
Parameter	Value	Parameter	Value
Burn time (s)	1.8	Radius (m)	0.0325
Dry mass (kg)	0.4938	Structural mass (kg)	1.079
Grain density (kg/m${}^{3}$)	1730	$I_{xx}=I_{yy}$ (kg m${}^{2}$)	0.1163
Outer radius (m)	0.022	$I_{zz}$ (kg m${}^{2}$)	0.0005
Inner radius (m)	0.003625	Drag coefficient	0.43
Grain height (m)	0.125	Nose length (m)	0.224
Number of grains	1	Nose type	von Kármán
Nozzle exit radius (m)	0.010	Number of fins	4
Throat radius (m)	0.005	Fin span (m)	0.105
Axial position (m)	−0.600	Root chord (m)	0.099
		Tip chord (m)	0.057
		Fin axial position (m)	−0.497

Displaying all individual sensor signals is not practical within the manuscript; however, the complete synthetic datasets are provided in the repository as CSV files:

**Table 6:** Virtual sensor configuration used for RocketPy data emulation.

Sensor	Range	Sampling rate (Hz)	Resolution	Noise density
ADXL375 (Accel.)	±200 g	24.98	16-bit	5 mg/$\sqrt{\text{Hz}}$
ICM42670 (Accel.)	±16 g	12.5	16-bit	100μg/$\sqrt{\text{Hz}}$
ICM42670 (Gyro)	±250 dps	12.5	16-bit	7 mdps/$\sqrt{\text{Hz}}$
MS5607 (Baro.)	200 000 Pa	12.5	24-bit	0.024 Pa

• ADXL375 accelerometer - RocketPy_Simulated_ADXL375_data.csv
• ICM42670 accelerometer - RocketPy_Simulated_ICM42670_A_data.csv
• ICM42670 gyrometer - RocketPy_Simulated_ICM42670_G_data.csv
• MS5607 barometer - RocketPy_Simulated_MS5607_data.csv

Once formatted according to the sensor protocols described in Section 6.1.2, these datasets are injected into the onboard computer through the SPI-based HIL platform at 12.5 Hz. This ensures that the full acquisition and processing chain operates under the same timing and interface constraints expected in real flight.

**Fig. 14:**
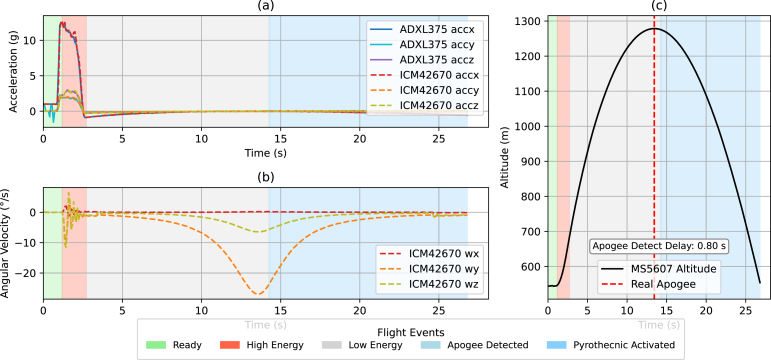
HIL flight simulation results. (a) Acceleration. (b) Angular velocity. (c) Altitude.

The resulting measurements extracted from the onboard computer after the HIL simulation are shown in Fig. 14. Unlike the RocketPy ground-truth data, these signals correspond to sensor-level measurements processed by the embedded system. As such, acceleration includes the gravitational component and is expressed in the sensor reference frame, while altitude is derived from barometric pressure and therefore referenced to the ambient pressure at the launch site, including any initial offset. The plots illustrate the evolution of acceleration, angular velocity, and altitude throughout the simulated mission, together with the corresponding state transitions identified by the onboard logic.

Prior to liftoff, the system remains in the READY state, with all subsystems initialized and the sensor suite actively sampling while the vehicle is assumed to be on the launch rail. The transition to powered flight is clearly reflected by a sharp and sustained increase in longitudinal acceleration, marking the thrust-dominated phase. Following motor burnout, the acceleration rapidly decreases, indicating the transition to a drag-dominated coasting regime. Apogee is identified near the point where the barometric altitude reaches its maximum and the pressure trend reverses, indicating the onset of descent. The descending portion of the trajectory corresponds to free-fall motion, as parachute deployment is not included in the present simulation and is reserved for future work. Overall, the consistency between the expected physical flight phases and the detected state transitions confirms that the onboard system correctly interprets sensor data and reproduces the intended flight-state evolution under realistic conditions.

The close agreement between injected synthetic data and recorded measurements confirms the correct operation of the acquisition chain, filtering routines, and embedded processing pipeline. All datasets were successfully retrieved via the LabVIEW interface without corruption, validating the reliability of the flash memory subsystem and the post-flight extraction workflow.

To quantitatively assess event-detection performance, additional HIL simulations were conducted under perturbed conditions. The corresponding plots are omitted for brevity, as their profiles remain consistent with the nominal case. Table 7 summarizes the evaluated scenarios and reports the apogee detection delay in each case, defined as the time difference between the simulated ground-truth apogee and the detection instant reported by the onboard system.

Each scenario introduces a controlled deviation from nominal flight conditions, affecting the trajectory and sensor signals in distinct ways. Variations in mass and drag primarily modify the vehicle’s energy balance, leading to changes in apogee and descent dynamics, while propellant variations alter the thrust profile and ascent behavior. In contrast, turbulent wind profiles introduce fluctuations in the measured signals without significantly altering the underlying trajectory. Together, these perturbations challenge the robustness of the onboard detection algorithms under both deterministic and stochastic disturbances.

Across all tested conditions, the detection delay remained below 1 s, demonstrating consistent timing performance even under significant deviations from nominal conditions. This delay is acceptable for small experimental sounding rockets because, after apogee, the vehicle rapidly transitions to gravity-dominated free fall, leading to a continuous increase in descent velocity and kinetic energy. Consequently, longer detection delays would result in higher deployment loads on the recovery system, whereas sub-second delays limit this effect and remain within typical structural and parachute deployment tolerances for this class of vehicle.

**Table 7:** Apogee detection delay under varying simulation parameters in HIL setup.

Parameters	Apogee detect delay
Standard conditions	0.80 s
Turbulent wind profile	0.88 s
Increased rocket mass (＋50%)	0.72 s
Increased drag coefficient (＋50%)	0.80 s
Combined mass and drag increase and turbulent wind	0.72 s
Reduced propellant mass (−50%)	0.72 s

### 7.1 Conclusions

The results obtained across multiple HIL campaigns demonstrate that the proposed framework enables a quantitative and system-level validation of the onboard computer without flight experiments. By combining a deterministic embedded architecture with physics-based trajectory generation and protocol-level sensor emulation, the approach ensures that the complete acquisition, processing, and decision-making chain is exercised under realistic timing and excitation conditions. This allows the onboard computer and its embedded algorithms to be evaluated under mission scenarios that significantly deviate from nominal assumptions, while maintaining full repeatability and control over test conditions.

Beyond validating a specific implementation, the proposed HIL framework constitutes a general platform for the development, verification, and comparison of onboard algorithms. In particular, it enables systematic evaluation of different flight-event detection strategies under identical and controlled conditions, including threshold-based approaches, model-based methods, and state-estimation techniques such as Kalman filtering. This capability is especially relevant for academic and experimental rocketry, where access to flight data is limited and costly, and where iterative algorithm development is often constrained by the availability of launch campaigns.

The validation results confirm that the onboard computer achieves reliable operation across all tested scenarios, including variations in aerodynamic properties, propulsion characteristics, and environmental disturbances. The observed sub-second apogee detection delays demonstrate that the embedded algorithms maintain consistent performance even under significant deviations from nominal conditions, while remaining compatible with the physical constraints imposed by recovery-system deployment dynamics.

In addition to event detection, the HIL campaigns verified all subsystems contributing to mission-critical functionality, including SPI communication with sensors, interrupt-driven acquisition routines, flash-memory management, pyrotechnic command logic, and post-flight data retrieval. By validating the complete sensing and actuation pipeline in a closed-loop experimental setup, the proposed approach provides a level of system verification that would otherwise require multiple flight tests.

Although a full flight campaign has not yet been conducted, the HIL results indicate a level of functional maturity of the onboard computer typically achieved only after repeated launches. The ability to reproduce complete mission timelines, execute the entire acquisition and processing pipeline, and validate flight-event detection against six-degree-of-freedom ground truth significantly reduces development risk while accelerating iterative design cycles.

Despite these advantages, the inherent limitations of the HIL-based validation approach must be recognized. The accuracy of the evaluation depends on the fidelity of the trajectory model and sensor emulation, which may not fully capture real-flight disturbances such as structural vibrations, aerodynamic uncertainties, and recovery-system dynamics. In particular, phenomena involving complex fluid–structure interactions or deployment transients remain difficult to reproduce in a purely simulation-based environment. Therefore, while the HIL framework provides a powerful and scalable tool for system verification and algorithm development, it complements rather than replaces experimental flight validation.

Based on the development and validation results, the main capabilities and current limitations of the proposed system can be summarized as follows:

• Deterministic acquisition and processing of multi-sensor data under realistic flight dynamics;
• End-to-end validation of the complete sensing, processing, and decision-making chain under realistic timing constraints;
• Reliable detection of flight events with sub-second delay under nominal and perturbed conditions;
• Capability to evaluate and compare onboard algorithms (e.g., threshold-based, model-based, and state-estimation methods) under controlled and repeatable conditions using the HIL framework;
• Robust non-volatile data logging and post-flight data extraction without corruption;
• Native support for hardware-in-the-loop validation through a dedicated sensor-emulation interface;
• Access to ground-truth reference trajectories enabling quantitative assessment of onboard estimation and detection performance;
• Modular hardware and software architecture that enables integration of additional sensors, recovery strategies, and mission profiles;
• Reduced dependence on launch campaigns for early-stage validation, lowering cost and operational risk in academic environments;
• Dependence on external interfaces (e.g., LabVIEW), although alternative automation pipelines can be implemented for simulations, device programming, and large-scale Monte Carlo campaigns;
• Limited modeling of recovery dynamics (e.g., parachute deployment) in the current HIL framework, representing a planned direction for future development;
• Absence of magnetometer emulation in the current implementation due to limitations of the trajectory simulator, though integration is straightforward for future studies.

## CRediT authorship contribution statement

**Nathan Andreani Netzel:** Writing – original draft, Validation, Software, Methodology, Investigation, Formal analysis, Data curation. **Leonardo Gabriel Rosa:** Software, Methodology, Investigation, Formal analysis, Data curation. **Francisco Granziera Jr.:** Writing – original draft, Supervision, Funding acquisition, Conceptualization. **Marcelo Carvalho Tosin:** Writing – review & editing, Supervision, Resources, Project administration, Funding acquisition, Conceptualization. **Daniel Strufaldi Batista:** Writing – review & editing, Writing – original draft, Validation, Supervision, Investigation, Conceptualization.

## Declaration of competing interest

The authors declare that they have no known competing financial interests or personal relationships that could have appeared to influence the work reported in this paper.

## Footnotes

1 **Safety considerations:** The procedures described above summarize the avionics configuration sequence but do not replace comprehensive launch-safety protocols. Once pyrotechnic devices are connected, strict safety measures must be enforced, including physical arming control, clear communication among team members, maintenance of safe separation distances, and the use of appropriate personal protective equipment (PPE). Detailed range-safety procedures are beyond the scope of this work but are mandatory in any experimental rocketry activity.

## References

[1] Vasant G., Suresh B. (2009). History of rocketry in India. Acta Astronaut..

[2] Migliorino M.T., Aiello M., Berti M., Rotondi M., D’Alessandro S., Bianchi D., Jahjah M., Pizzarelli M. (2022). Student firing tests and launches with commercial and self-made solid rocket motors. Acta Astronaut..

[3] R. Lenormand, A. Baras, T. Aujames, A. Lacombe, J. Delpeyrat, Successful collaboration knowledge transfer amongst student space clubs - the SERA rockets, in: Proceedings of the 8th European Conference for Aeronautics and Space Sciences, 2019.

[4] Kobald M., Fischer U., Tomilin K., Petrarolo A., Schmierer C. (2018). Hybrid experimental rocket stuttgart: A low-cost technology demonstrator. J. Spacecr. Rockets.

[5] Bohrer T.W. (2017).

[6] C. Bach, J. Sieder, A. Konietzke, M. Tajmar, Sounding rocket development with liquid propellants within the dlr stern programme, in: 1st Symposium on Space Educational Activities, Italy, 2015.

[7] A. Stamminger, H. Ciezki, W.H. Kitsche, M. Kobald, K. Lappöhn, A. Schmidt, STERN – A Rocket programme for German Students, in: 21st ESA Symposium on European Rocket & Balloon Programmes and Related Research, Thun, Switzerland, 2013, 2013.

[8] Dupont C., Bullock M., Prevost M., Bec R., Maisonneuve Y., Pillet N., Barenes R. (2006). 42nd AIAA/ASME/SAE/ASEE Joint Propulsion Conference & Exhibit.

[9] Tosin M.C., Granziera Júnior F., Chiozzo M.F., Gibim F., Gazzoni Filho D.L., Deliberador A., Rigon E. (2003).

[10] H. Hingre, J. Oswald, A. Galéon, PERSEUS - European Space Research Program for Students, in: 6th European Conference for Aeronautics and Space Sciences, 2015.

[11] German Aerospace Center (DLR) (2026). https://hyend.de/index.php/dlr-stern-participation/.

[12] Delft Aerospace Rocket Engineering (2026). https://en.wikipedia.org/wiki/Delft_Aerospace_Rocket_Engineering.

[13] Hellmann H., Persson O., Stamminger A., Schmidt A. (2009). Proceedings of the 19th ESA Symposium on European Rocket and Balloon Programmes and Realted Research.

[14] J. Martens, X. Cui, L. Alacoque, T. Messinger, A. Hamilton, W. Van der Meulen, A. Deshpande, Student Organization for Aerospace Research Atlantis II. Sounding Rocket, SA CUP Project Technical Report, 2018.

[15] Spearrin R.M., Bendana F.A. (2019). Design-build-launch: a hybrid project-based laboratory course for aerospace engineering education. Acta Astronaut..

[16] Osborn R. (2026). https://www.gbnet.net/~richard/rocketry/altchart.html.

[17] MissileWorks (2026). https://www.missileworks.com/rrc3.

[18] PerfectFlite (2026). http://www.perfectflite.com/SLCF.html.

[19] Featherweight Altimeters (2026). https://www.featherweightaltimeters.com/raven-altimeter.html.

[20] Bdale Garbee (2026). https://altusmetrum.org/TeleMini/.

[21] Tosin M.C., Granziera F., Gibim F., Deliberador A., Rigon E. (2004).

[22] STMicroelectronics (2024). https://www.st.com/resource/en/datasheet/dm00037051.pdf.

[23] Devices A. (2013). https://www.analog.com/media/en/technical-documentation/data-sheets/ADXL375.PDF.

[24] Filho A.G., Rosa L.G., Batista D.S., Granziera F., Tosin M. (2024). Anais do XII Congresso Nacional de Engenharia Mecânica.

[25] InvenSense T. (2022). https://invensense.tdk.com/wp-content/uploads/2021/07/ds-000451_icm-42670-p-datasheet.pdf.

[26] Connectivity T. (2020). https://www.te.com/commerce/DocumentDelivery/DDEController?Action=srchrtrv%26DocNm=MS5607-02BA03%26DocType=Data%20Sheet%26DocLang=English%26DocFormat=pdf%26PartCntxt=MS560702BA03-50.

[27] MEMSIC (2019). https://www.memsic.com/Public/Uploads/uploadfile/files/20220119/MMC5983MADatasheetRevA.pdf.

[28] Renesas (2023). https://www.renesas.com/en/document/dst/at45db641e-datasheet?r=1608921.

[29] Technology M. (2019). https://ww1.microchip.com/downloads/aemDocuments/documents/OTH/ProductDocuments/DataSheets/MIC2915x-30x-50x-75x-High-Current-Low-Dropout-Regulators-DS20005685B.pdf.

[30] Instruments T. (2025). https://www.ti.com/lit/ds/symlink/lp5907.pdf.

[31] Ceotto G., Carmo B., Schmitt R., Pezente L., Fernandes Alves G. (2021). RocketPy: Six degree-of-freedom rocket trajectory simulator. J. Aerosp. Eng..

[32] Batista D.S., Granziera F., Tosin M.C., Melo L.F. (2023). Attitude-independent magnetometer calibration using nonlinear least squares. IEEE Sens. J..

[33] Netzel N.A., Silva F.O.e., Tosin M.C. (2025). Proceedings of the 3rd Brazilian Aerospace Congress.

[34] Mohandoss T., R. F. (2024). Vibration analysis and electrical contact resistance assessment for automotive relays. J. Eng. Sci. Technol. Rev..

[35] L. Pepermans, E. Menting, M. Rozemeijer, B. Koops, N.S. Dahl, N. Suard, S. Khurana, F.V. Marion, F. Kuhnert, M. Serman, Comparison of Various Parachute Deployment Systems for Full Rocket Recovery of Sounding Rockets, in: Proceedings of the 8th European Conference for Aeronautics and Space Sciences, Madrid, Spain, 2019, 10.13009/EUCASS2019-411.

[36] Jasztal M., Kłosiński A. (2024). Design and application of a parachute deployment mechanism for sounding rockets based on commonly available and affordable components. J. Konbin.

[37] Netzel N.A., Batista D.S., Granziera F., Tosin M.C. (2025). A versatile low-cost data acquisition system for small rocket engine test bench. HardwareX.

[38] Krepski G.G., Garbelini Filho A., Barros C.M., Rosa L.G., Daefiol M.A., Netzel N.A., Gibim F., Batista D.S., Granziera F., Tosin M.C. (2025). 3°Congresso Aeroespacial Brasileiro.

